# Systematic review of analytical methods applied to longitudinal studies of malaria

**DOI:** 10.1186/s12936-019-2885-9

**Published:** 2019-07-29

**Authors:** Christopher C. Stanley, Lawrence N. Kazembe, Mavuto Mukaka, Kennedy N. Otwombe, Andrea G. Buchwald, Michael G. Hudgens, Don P. Mathanga, Miriam K. Laufer, Tobias F. Chirwa

**Affiliations:** 1 School of Public Health, Faculty of Health Sciences, University of the Witwatersrand, Johannesburg, South Africa; 2 Malaria Alert Centre, University of Malawi College of Medicine, Blantyre, Malawi; 3 Department of Statistics, University of Namibia, Windhoek, Namibia; 4 Oxford Centre for Tropical Medicine and Global Health, Oxford, UK; 5 Mahidol-Oxford Tropical Medicine Research Unit, Bangkok, Thailand; 6 Perinatal HIV Research Unit, Faculty of Health Sciences, University of the Witwatersrand, Johannesburg, South Africa; 7 Center for Vaccine Development and Global Health, University of Maryland School of Medicine, 685 W. Baltimore St. HSF-1 Room 480, Baltimore, MD 21201 USA; 8 Department of Biostatistics, Center for AIDS Research, University of North Carolina Chapel Hill, Chapel Hill, NC USA

**Keywords:** *Plasmodium falciparum*, Longitudinal studies, Longitudinal analysis, Cohort studies

## Abstract

**Background:**

Modelling risk of malaria in longitudinal studies is common, because individuals are at risk for repeated infections over time. Malaria infections result in acquired immunity to clinical malaria disease. Prospective cohorts are an ideal design to relate the historical exposure to infection and development of clinical malaria over time, and analysis methods should consider the longitudinal nature of the data. Models must take into account the acquisition of immunity to disease that increases with each infection and the heterogeneous exposure to bites from infected *Anopheles* mosquitoes. Methods that fail to capture these important factors in malaria risk will not accurately model risk of malaria infection or disease.

**Methods:**

Statistical methods applied to prospective cohort studies of clinical malaria or *Plasmodium falciparum* infection and disease were reviewed to assess trends in usage of the appropriate statistical methods. The study was designed to test the hypothesis that studies often fail to use appropriate statistical methods but that this would improve with the recent increase in accessibility to and expertise in longitudinal data analysis.

**Results:**

Of 197 articles reviewed, the most commonly reported methods included contingency tables which comprised Pearson Chi-square, Fisher exact and McNemar’s tests (n = 102, 51.8%), Student’s t-tests (n = 82, 41.6%), followed by Cox models (n = 62, 31.5%) and Kaplan–Meier estimators (n = 59, 30.0%). The longitudinal analysis methods generalized estimating equations and mixed-effects models were reported in 41 (20.8%) and 24 (12.2%) articles, respectively, and increased in use over time. A positive trend in choice of more appropriate analytical methods was identified over time.

**Conclusions:**

Despite similar study designs across the reports, the statistical methods varied substantially and often represented overly simplistic models of risk. The results underscore the need for more effort to be channelled towards adopting standardized longitudinal methods to analyse prospective cohort studies of malaria infection and disease.

**Electronic supplementary material:**

The online version of this article (10.1186/s12936-019-2885-9) contains supplementary material, which is available to authorized users.

## Background

*Plasmodium falciparum* infection is one of the most common parasitic infection in humans. Individuals in high-transmission settings are exposed to bites of infected *Anopheles* mosquitoes and develop frequent infections. In early childhood, infections are almost always associated with symptomatic disease [1]. Over time, individuals acquire immunity to clinical disease and, to some degree, infection [2, 3]. Thus, each infection alters the host immune response that protects against the next episode. In addition, risk of exposure to infected bites is not uniformly distributed [4, 5]. Thus, risk of infection is not equally distributed across populations. While one infection may decrease the risk of disease or infection, an infection in an individual is also an indicator of more frequent exposure to infected mosquitoes. This provides an interesting challenge to modelling the risk of malaria disease and infection over time. Longitudinal cohorts, unlike other study designs such as cross-sectional surveys, can differentiate between infections that appear asymptomatic at time of detection but may become symptomatic over time. With efforts intensified to prevent *Plasmodium* infection and reduce clinical malaria disease, prospective longitudinal cohorts are increasingly being conducted to assess risk of infection and disease over time.

Well-designed prospective longitudinal cohort studies can be key to understanding risk of malaria disease for participants over time, but appropriate analysis of the collected data is also critical for accurate results. Like any longitudinal designs, malaria cohorts are characterized by repeated measurements, resulting in possible correlated infection and disease episode data from same participant. The underlying assumption of no correlation among observations made by standard statistical methods such as t-tests, analyses of variance (ANOVA), and linear regression are violated [6–8]. The use of inappropriate methods, particularly those that do not account for possible correlation of events when dealing with repeated episodes of malaria or infection, can lead to biased results. Ignoring correlation of events could result in the confidence intervals for the estimated rates being artificially narrow and the null hypothesis may be rejected more often than warranted [9, 10]. Appropriate longitudinal data analysis techniques should account for such possible within-participant correlation and different covariance structures of episodes of malaria or infection measurements over time. These include generalized estimating equations (GEE) and mixed-effects models. The superiority in consistency and efficiency of these methods over the traditional methods, such as ANOVA, t-tests and simple linear regression were demonstrated previously [11–14]. One of the attractive features of GEE is that the method is robust to mis-specification of the correlation structure, such that the estimator is consistent even if the working correlation structure is mis-specified [15], although it can be inefficient under the mis-specified working correlation structure [16]. When the research interest is on repeated time-to-episodes of disease or infection, it is common to analyse time-to-first episode ignoring the subsequent events, but this approach fails to utilize all information available in the data [13, 17–19]. Analyses of time-to-first event only may be preferred in some situations. For example, when the intention is to evaluate interventions or prognostic factors (e.g., some vaccines) as ‘all-or-nothing’ determinants of malaria risk, so occurrence of any events is considered a failure. Another scenario would be when the first event is considered to have ‘reset’ the host such that subsequent events may then be consequently considered in a different category. Recurrent time-to-event models are the appropriate techniques for analysing recurrent malaria episode data because they take into account the within-participant non-independence of episodes. These techniques include frailty models [20, 21] or recurrent Cox-extended models, such as the Andersen–Gill and Prentice–Williams–Peterson models [17].

The statistical models that incorporate all the repeated observations over follow-up are particularly important in malaria since over time, malaria infections result in individuals acquiring immunity to malaria disease. Each infection may introduces protective effect to future disease episodes. Therefore, appropriate analytical methods for repeated episodes of disease and infection should capture the impact of the developed protective immunity.

Recent advancements in statistical methods and computer software have improved the ease of access and application of statistical methods specifically designed for longitudinal studies, allowing one to handle complexity with cohort data [9, 22–25]. Limited information is available about whether the recent developments in computer software and statistical methods for longitudinal data has translated into more wide adoption and application of appropriate methods when analysing prospective longitudinal malaria cohort data. This study tested the hypotheses that choice of statistical methods would change over time and that these changes would reflect more appropriate analytical methods. To address this hypothesis, a systematic review was conducted to investigate the trends of use of appropriate statistical methods among articles analysing prospective longitudinal cohort data for clinical malaria episodes or *Plasmodium* infection published over a 22-year period.

## Methods

### Search strategy

Titles and abstracts containing the terms “malaria prospective study” or “malaria cohort study” or “malaria longitudinal study” or “malaria years follow-up” or “malaria repeated measurements” indexed in PubMed, Medline or ScienceDirect were included in the search. Original articles in English, which was a familiar language to authors, published from 1996 until December 2017 were reviewed in chronological order.

### Inclusion and exclusion criteria

**Fig. 1:**
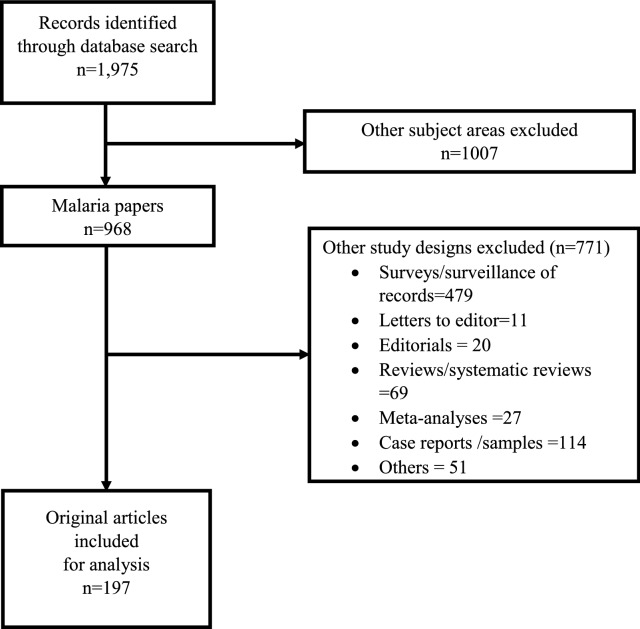
Flow chart for study screening process of articles

Original research articles were included in the analysis if they were prospective cohort studies where participants were actively or passively followed up for repeated malaria episodes over time and described the following outcomes: clinical malaria based on clinical symptoms and confirmed by rapid diagnostic test (RDT) or microscopy, infection defined as polymerase chain reaction (PCR), microscopy, or RDT results positive for *Plasmodium* parasites. Several publications reporting on the same study or data set were all included. Letters to the editor, editorials, reviews or systematic reviews, meta-analyses, repeated cross-sectional surveys, case reports and articles written in languages other than English were excluded. Detailed selection process of the studies under review is shown in Fig. 1.

### Data extraction

For each article included in the analysis, the following information was extracted: year of publication, duration of follow-up, outcomes, sample size, population, location and type of statistical methods used. Statistical methods were grouped according to categorization by Colditz and Emerson [26, 27] as presented in Appendix. In the cases where researchers studied multiple outcomes, only methods applied to the outcomes of interest-clinical malaria or infection—were considered. Particular interest was on standard longitudinal analysis techniques including GEE, mixed-effects models (random effects models) and repeated measures ANOVA which are considered appropriate for analyses of repeatedly measured data such as repeated episodes malaria disease or infection. For repeated time-to-episodes, recurrent Cox-based models and frailty models are considered appropriate, while other survival methods such as the Kaplan–Meier estimator, log-rank test and Cox model are only appropriate for analyses of time-to-single-episode.

### Statistical analysis

Logistic GEE was used to estimate the odds of using the types of statistical methods in the 2007–2017 period compared to 1996–2006 period. Each model adjusted for study sample size and year of publication was included as a panel variable. The outcome variable was binary, indicating whether in each article a particular method was used or not. Models were constructed for each of the following methods: contingency tables, descriptive statistics only, Kaplan–Meier estimator, Cox model, Poisson model, GEE and mixed-effects model. All analyses were done using Stata SE version 15.1 (Stata Corp, College Station, TX, USA).

## Results

**Fig. 2:**
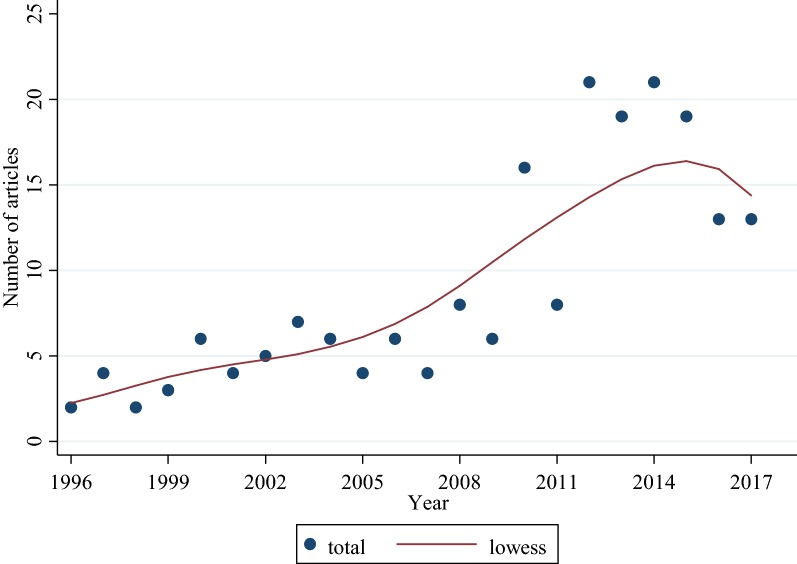
Total number of articles from 1996 to 2017 with lowess curve. The number of articles published increased over the 22-year study period, with the highest number found between 2012 and 2016

Out of the 1975 abstracts reviewed, 1778 were excluded because they were of other subject areas (n = 1007, 51.0%) and study designs (n = 771, 39.0%). One-hundred and ninety-seven (10.0%) articles met the inclusion criteria and were included in the analyses (Fig. 1). Overall, the median follow-up time was 12 months [interquartile range (IQR): 9–24]. The median sample size per article was 351 (IQR: 206–700). The number of articles increased over the 22-year study period, with the highest number found between 2012 and 2016 (Fig. 2). Overall, there were 128 (65.0%) articles that analysed both clinical malaria episodes and infection as outcomes; 53 (26.9%) assessed clinical malaria episodes only, and 16 (8.1%) infections only. A table listing articles reviewed in this study with details of year of publication, duration of follow-up, outcomes, sample size, population, location, and analysis methods are provided as Additional file 1: Table S1.

Among 197 articles reviewed, the most commonly reported methods included contingency tables comprising Pearson Chi-square, Fisher exact and McNemar’s tests (n = 102, 51.8%), followed by Student’s t-tests (n = 82, 41.6%), multiple linear regression (n = 71, 36.0%), Cox models (n = 62, 31.5%), and Kaplan–Meier estimators (n = 59, 30.0%) (Table 1). The frequency of usage of different statistical methods changed between the two study periods: 1996–2006 versus 2007–2017. The proportion of articles using contingency tables was higher in the first period than in the second, 65.3 versus 47.3% as was simple linear regression, 10.2 versus 0.7%. Use of the survival analysis methods, Cox models and Kaplan–Meier estimators increased in the second period from 18.4 to 35.8% and 22.5 to 32.4%, respectively. Similarly, usage of mixed-effects models among longitudinal data analysis techniques was higher in the later period compared to the former, 4.1 versus 14.9%.

**Table 1:** Frequency of articles using a particular statistical method

Method	All (n = 197)	1996–2006 (n = 49)	2007–2017 (n = 148)
	N (%)	N (%)	N (%)
Descriptive statistics only	17 (8.6)	5 (10.2)	12 (8.1)
Contingency tables	102 (51.8)	32 (65.3)	70 (47.3)
Multiway tables	50 (25.4)	16 (32.7)	34 (23.0)
Epidemiologic statistics	37 (18.8)	12 (24.5)	25 (16.9)
t-tests	82 (41.6)	23 (46.9)	59 (39.9)
Analysis of variance	18 (9.1)	1 (2.0)	17 (11.5)
Simple linear regression	6 (3.1)	5 (10.2)	1 (0.7)
Non-parametric tests	48 (24.4)	11 (22.5)	37 (25.0)
Non-parametric correlation	21 (10.7)	3 (6.1)	18 (12.2)
Multiple regression	71 (36.0)	15 (30.6)	56 (37.8)
Poisson regression	49 (24.9)	8 (16.3)	41 (27.7)
Negative binomial regression	22 (11.2)	3 (6.1)	19 (12.8)
Survival analysis			
Kaplan–Meier estimator	59 (30.0)	11 (22.5)	48 (32.4)
Log-rank test	36 (18.3)	7 (14.3)	29 (19.6)
Cox model	62 (31.5)	9 (18.4)	53 (35.8)
Other survival methods	4 (2.0)	3 (6.1)	1 (0.7)
Longitudinal analysis			
Generalized estimating equations	41 (20.8)	9 (18.4)	32 (21.6)
Mixed-effects regression	24 (12.2)	2 (4.1)	22 (14.9)
Recurrent analysis			
Shared frailty model	2 (1.0)	–	2 (1.4)
Andersen–Gill (AG) model	4 (2.0)	–	4 (2.7)

From the 197 articles included in the review, 131 (66.5%) analysed repeated malaria episodes or infection, of which 60 (45.8%) used appropriate standard longitudinal analysis methods. Appropriate longitudinal analyses included 36 (60.0%) instances of GEE, 19 (31.7%) articles employing mixed-effects models, and 5 (8.3%) articles utilizing both the GEE and mixed-effects models. Seventy-one (54.2%) of the 131 articles used other methods, namely the Poisson (n = 49) and negative binomial regression models (n = 22). These models were based on the generalized linear modelling. Of the 41 studies that modelled the correlation structure, only 5 (12.2%) assumed exchangeable correlation, while 36 (87.8%) did not specify the correlation structure. Out of all 197 articles reviewed, there were 99 (50.3%) studies that assumed Poisson distribution, 4 of these assessed overdispersion, of which 2 adjusted for it. In 9 (4.7%) of all the 197 reviewed studies, authors assumed a normal distribution of count data without indicating if transformations were done.

There were 74 of 197 articles that analysed time-to-first malaria episode or infection ignoring subsequent events and 61 (82.4%) of these employed survival analysis techniques that included Cox models, Kaplan–Meier estimators, and log-rank tests. There were 24 (12.2%) of 197 articles that analysed repeated time-to-episodes and only 6 (25.0%) of these used appropriate recurrent event models with extensions of Cox model, namely Andersen–Gill (n = 4) and frailty models (n = 2) to evaluate risk factors for recurrent clinical malaria. Eighteen (9.1%) articles analysed repeated time-to-malaria or infection episodes using Cox models and Kaplan–Meier estimators which are only valid for single-event analyses because they assume independence of events.

**Fig. 3:**
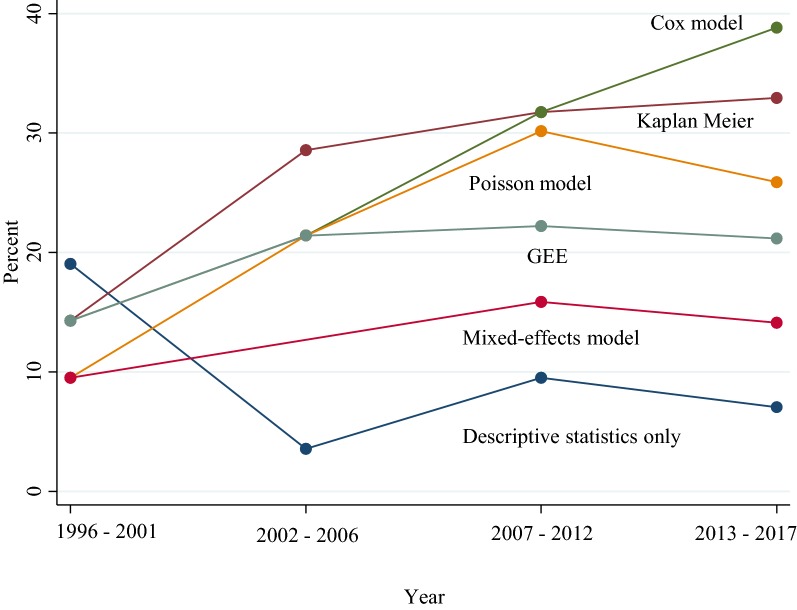
Time series plot with percentage of articles using a particular statistical method, from 1996 to 2017. The percentage of articles using Cox models, Kaplan–Meier estimators increased over entire period while GEE, and mixed-effects models increased before remaining stable

Compared to the first period 1996–2006, the odds of an article using contingency tables adjusting for sample size were lower in the second period 2007–2017 [odds ratio (OR) = 0.49, 95% confidence interval (CI) 0.27–0.91] (Table 2). The proportion of articles using Cox models, Kaplan–Meier estimators and mixed-effects models increased over time (Fig. 3). However, only the Cox models, and mixed-effects models had increased odds of being used in the second period compared to the first period, (OR = 2.57, 95% CI 1.21–5.42) and (OR = 3.85, 95% CI 1.13–6.39), respectively (Table 2).

**Table 2:** Odds ratio of using a statistical method during 2007–2017 compared to 1996–2006 adjusted for article sample size

Method	Odds ratio (OR)	95% CI
Descriptive statistics only	0.81	0.26–2.42
Contingency table	0.49	0.27–0.91
Kaplan–Meier estimator	1.80	0.88–3.67
Cox model	2.57	1.21–5.42
Log-rank	1.60	0.66–3.84
Poisson model	1.78	0.85–3.72
GEE	1.16	0.56–2.39
Mixed-effects model	3.85	1.13–6.39

*CI* confidence interval

## Discussion

In this review of statistical methods among articles analysing prospective longitudinal cohort data for clinical malaria episodes or *Plasmodium* infection published over a 22-year period, the statistical methods substantially varied across articles despite that they reported analysis of the same general study design and outcome measurements. The most commonly used models for analysing count number of malaria episodes included the Poisson and negative binomial models, but these fall short with repeated measurements data as they cannot account for within-participant correlation of the episodes [6, 28, 29]. Ignoring this possible correlation of events tends to bias the model results as demonstrated previously [15, 30–32], yielding regression parameters with underestimated standards errors for time-dependent covariates or overestimated errors in time-dependent covariates [33]. As hypothesized, trends in choice of appropriate methods improved over time, which included the GEE and mixed-effects models as well as recurrent event models, although their usage during the entire study period remained low. Furthermore, the majority of the studies that modelled the correlation structure did not specify the kind of assumed correlation structure, with only exchangeable correlation structure stated in few studies. Among studies that assumed Poisson distribution, the majority of them did not state whether or not overdispersion was checked.

Some articles analysed the repeated time-to-disease episodes or infections but applied the Cox model [34] or the Kaplan–Meier estimator, which assume independent events and are only valid for modelling the time-to-single disease episode or infection. Such analyses may yield biased results. This is particularly true in malaria because over time, individuals acquire immunity to clinical disease and, to some degree, infection. Therefore, each infection may be dependent on the past, and in turn alter the host immune response that protects against the next episode. Additionally, there may be temporal variations in exposure which can alter the risk of clinical malaria disease over time. In some instances, data from the later episodes were discarded leading to loss of information which may limit the resulting conclusions. Modelling that focuses on time-to-first event only may not be generalizable to analyses examining all repeated episodes of disease or infection over entire study period because the risk to subsequent episodes diminishes as a result of the gained immunity over time.

When analysing repeated times-to-episodes malaria or infection, analytical methods should account for possible within-participant correlation of events as well as censoring over follow-up. Such appropriate methods include recurrent event survival models [35, 36]. In the current review, only six articles analysed repeated time-to-episodes using appropriate recurrent models. Well-developed descriptions of the recurrent event models have been presented by several authors [10, 17, 35, 36] while this section briefly discusses these models in relation to malaria data. The recurrent event models include extensions of the Cox model such as Andersen–Gill (AG) [37], Prentice–Williams–Peterson (PWP) [38] and the frailty model [39]. The AG model uses a counting process time-scale for all episodes assuming that the correlation between episodes-times for each participant is explained by previous episodes where time-scale does not reset to zero after an episode. This model is typically applicable when the interest is on the overall effect of covariates on the intensity of the recurrent episodes. The PWP model analyses ordered events by stratification and can be expressed as gap-time model where the time-scale is reset to zero after each episode occurs. Gap times between malaria parasitaemia detection to clearance is a good example where the PWP model can be valid. Moreover, the PWP model can give both overall and episode-specific effects and so can be more applicable in malaria where covariate effects may be different in subsequent episodes due to the naturally acquired immunity. The shared frailty model extends the Cox model by introducing a random covariate that induces dependence among the recurrent episode times. By conditioning on covariates and the random effect, the recurrent episodes are assumed to become independent. Another challenge when analysing recurrent events in malaria is the issue of exclusion of periods-at-risk after an event, and this may constrain the choice of statistical methods. Depending on kind of treatment if any, recurrent events within a few days of each other are likely to be the result of the same infection or the second event may be considered to be due to treatment failure. Ignoring periods-at-risk after an event is also a potential source of bias. Thus, authors ought to consider exclusion of periods-at-risk after an event during the datasets structuring before analyses are conducted.

This review has demonstrated that publications on prospective longitudinal malaria cohorts are increasing over time, a trend reflecting the growing understanding of the importance of longitudinal cohort data coupled with advancements in computer software developments for analysing such data [9, 22]. Optimal methods for longitudinal data analysis were in their infancy 20 years ago and both access to appropriate software and general acceptable of these methods by the research community have increased dramatically over this period. The increased trend in malaria cohort studies and the improved but slow adoption of appropriate methods underscores the need for standardized analytical methods for such data to avoid drawing erroneous conclusions based on inaccurate results.

This review may have included more than one report using the same data, which may have introduced bias due to duplication of datasets. For example, the same data may have been analysed using inappropriate methods in first instance and then using appropriate techniques. Furthermore, some authors published more than one article and such articles may share similarities in the choice of analytical methods, but accounting for this was beyond the scope of this review as author background information was not collected. Future studies should consider testing whether more appropriate statistical methods improve or change understanding of malaria epidemiology by using among other factors, authors’ background information. Lastly, further studies may explore and compare how results from this study would compare with trends specifically from field trials.

## Conclusions

Cohort studies assessing risk of malaria infection and disease over time did not employ consistent analytic methods. The statistical methods varied substantially and often represented overly simplistic models despite similar designs, which may have led to biased outcomes and results that cannot be compared across studies. This review suggests that recent studies are adopting more appropriate statistical methods, though the statistical analyses are not uniform. These results underscore the need for more effort to be channelled towards adopting standardized longitudinal methods to model recurrent events for malaria infection and disease.

## Additional file

**Additional file 1: Table S1.** List of reviewed articles in the study with details of year of publication, duration of follow-up, outcomes, sample size, population, location and analysis methods.

## Data Availability

All data generated and analysed during this study are included in additional files of this published article

## Appendix

See Table 3.

**Table 3:** Categories of statistical methods used to assess the statistical content of articles

Category	Brief description	Include
No statistical methods or descriptive statistics only	Describe basic features of data to provide simple measures of summaries	No statistical content, or descriptive statistics only e.g., percentages, means, standard deviations, standard errors, histograms
Contingency tables	Cross tabulations used to summarize the relationship between categorical data	Chi-square test, Fisher’s exact test, McNemar’s test
Epidemiologic statistics	Measures of association for outcome of interest such as disease and some exposure(s)	Relative risk, risk ratios, rate ratios, risk difference, rate difference, odds ratio, log odds, risk difference, attributable risk fraction, sensitivity, specificity
Multiway tables	Extend two-way relationships to include three or more variables	Mantel–Haenszel procedure, log-linear models, logistic regression
t-test	Assess mean differences between groups	One-sample, matched-pair, two-sample t-tests
Pearson’s correlation	Measures linear correlation between two variables	Classical product-moment correlation
Simple linear regression	Regression that summarizes relationships between two continuous variables, an explanatory and a response	Least-squares regression with one predictor and one response variable
Multiple regression	Extends the simple regression to include two or more explanatory variables for a response	Polynomial regression and stepwise regression
Analysis of variance	Assess within and between group differences in means	Analysis of variance, Analysis of covariance, simple linear contrasts, F-tests
Multiple comparisons	Methods for handling multiple inferences on same data sets	Bonferroni techniques, Scheffé’s contrasts, Duncan multiple-range methods, Newman–Keuls procedure
Non-parametric test	Tests used when data is not assumed to follow a particular distribution, and are based on ranks of data	Sign test, Wilcoxon signed-rank test, Mann–Whitney test, Kruskal–Wallis test, Friedman test, Kolmogorov–Smirnov test
Non-parametric correlation	Measure strength and direction of association between two variables	Spearman’s rho, Kendall’s tau, monotone regression, test for trend
Survival analysis	Methods where outcome variable is the time until the occurrence of an event	Actuarial life table, Kaplan–Meier estimator for survival, survival function, Cox model, other parametric survival models, rate adjustment, log-rank test, Breslow’s test
Sensitivity analysis	Examines sensitivity of outcome to small changes in parameters of model or in other assumptions	Sample size, multiple outcomes, model distribution assumptions
Transformation	Use of data transformation often in regression	Natural logarithm, square, cubic
Cluster analysis	Involves dividing a multivariate dataset into “natural” clusters (groups) for in-depth assessment	Hierarchical, K-means, two-step clustering
Repeated-measures analysis	Approaches that account for correlation for within-participant observations and non-constant variance in response over time	Generalized estimating equations (GEE), mixed-effects models, repeated measures ANOVA
Other	Other methods not specified in above	Receiver-operating characteristic, principal component analysis, power analysis, propensity score

## Abbreviations

AIDS: acquired immune deficiency syndrome
ANOVA: analysis of variance
GEE: generalised estimating equations
HIV: human immunodeficiency virus
PCR: polymerase chain reaction
RDT: rapid diagnostic test
