# Supplementary material for: Systematic review of analytical methods applied to longitudinal studies of malaria
Source: Malar J. 2019 Jul 29;18:254. doi: 10.1186/s12936-019-2885-9 (PMC6664716; doi:10.1186/s12936-019-2885-9)
Supplement: Supplementary file 1 — Additional file 1: Table S1. List of reviewed articles in the study with details of year of publication, duration of follow-up, outcomes, sample size, population, location and analysis methods. [file 12936_2019_2885_MOESM1_ESM.docx]

**Title: Systematic review of analytical methods applied to longitudinal studies of malaria**

**Authors:** Christopher C. Stanley,^1,2^ Lawrence N. Kazembe,^3^ Mavuto Mukaka,^4,5^ Kennedy N. Otwombe,^1,6^ Andrea G. Buchwald,^7^ Michael G. Hudgens,^8^ Don P. Mathanga,^2^ Miriam K. Laufer,^7^ Tobias F. Chirwa,^1^

**Institutional affiliations:** ^1^School of Public Health, Faculty of Health Sciences, University of the Witwatersrand, Johannesburg, South Africa; ^2^Malaria Alert Centre, University of Malawi College of Medicine, Blantyre, Malawi; ^3^Department of Statistics, University of Namibia, Windhoek, Namibia; ^4^Oxford Centre for Tropical Medicine and Global Health, Oxford, United Kingdom; ^5^Mahidol-Oxford Tropical Medicine Research Unit, Bangkok, Thailand; ^6^Perinatal HIV Research Unit, Faculty of Health Sciences, University of the Witwatersrand, Johannesburg, South Africa; ^7^Center for Vaccine Development and Global Health, University of Maryland School of Medicine, Baltimore, USA; ^8^Department of Biostatistics, Center for AIDS Research, University of North Carolina Chapel Hill, North Carolina, USA.

**Additional file 1: Table S1. List of reviewed articles in the study with details on year of publication, duration of follow-up, outcomes, sample size, population, location and analysis methods.**

| **Article number** | **Author** | **Title** | **Year of publication** | **Outcomes** | **Duration of follow up** | **Sample size** | **Population** | **Location** | **Analysis methods** |
| --- | --- | --- | --- | --- | --- | --- | --- | --- | --- |
| **1** | Luxemburger C et al | The epidemiology of malaria in a Karen population on the western border of Thailand | 1996 | Incidence of malaria episodes | 1 year | 249 | School children and adults | Thailand | Descriptives, student t test, Chi square test, Fisher exact test, Mann-Whitney U test, rate ratios |
| **2** | Steketee RW et al | Malaria parasite infection during pregnancy and at delivery in mother, placenta, and newborn: efficacy of chloroquine and mefloquine in rural Malawi | 1996 | Incidence of parasitemia | 9 months | 1528 | Pregnant women | Malawi | Descriptives, Fisher exact, Chi square test, student t test, multivariate regressions, odds ratio |
| **3** | al-Yaman F et al | Reduced risk of clinical malaria in children infected with multiple clones of Plasmodium falciparum in a highly endemic area: a prospective community study | 1997 | Repeated malaria episodes, parasitemia | 1 year | 236 | Children aged c 18 years | Papua New Guinea | Descriptives, Chi square test, Poisson regression |
| **4** | Maitland K et al | Absence of malaria-specific mortality in children in an area of hyperendemic malaria | 1997 | Incidence of clinical malaria | 19 months | 1232 | Children < 10 years old | Melanesia | Descriptives |
| **5** | al-Yaman F et al | Association between cellular response (IL-4) to RESA/Pf155 and protection from clinical malaria among Papua New Guinean children living in a malaria endemic area | 1997 | Incidence of malaria episodes, parasitemia | 1 year | 207 | Children aged 0.5-15 years | Papua New Guinea | Descriptives, Fisher exact test, histograms, incidence rates |
| **6** | Lemnge MM et al | Maloprim malaria prophylaxis in children living in a holoendemic village in north-eastern Tanzania | 1997 | Incidence of clinical malaria, parasitemia prevalence and density | 52 weeks | 249 | Children aged 1-9 years | Tanzania | Descriptives, student t test, chi square test, Fisher exact test |
| **7** | Luxemburger C et al | Clinical features cannot predict a diagnosis of malaria or differentiate the infecting species in children living in an area of low transmission | 1998 | Incidence of malaria episodes, parasitemia | 7 months | 1527 | Children aged 2-15 years | Thailand | Descriptives, Mann-Whitney U test, logistic regression, Fisher exact test, Chi square test, sensitivity and specificity |
| **8** | Wagner G et al | High incidence of asymptomatic malaria infections in a birth cohort of children less than one year of age in Ghana, detected by multicopy gene polymerase chain reaction | 1998 | Incidence of asymptomatic malaria infections | 1 year | 71 | New-borns in community | Ghana | Descriptives, Cox regression, Weibull regression, mixed-effects logistic regression, likelihood ratio test |
| **9** | Bloland PB et al | Longitudinal cohort study of the epidemiology of malaria infections in an area of intense malaria transmission II. Descriptive epidemiology of malaria infection and disease among children | 1999 | Malaria infection and disease | 2 years | 1848 | Children than 15 years of age | Kenya | Descriptives, generalised estimating equations |
| **10** | Rogier C et al | Plasmodium falciparum clinical malaria in Dielmo, a holoendemic area in Senegal: no influence of acquired immunity on initial symptomatology and severity of malaria attacks | 1999 | Incidence of malaria episodes, parasitemia, time to infection recovery | 3 years | 226 | Children and adults in community | Senegal | Descriptives, Kaplan Meier estimator, Mann-Witney U test, mixed-effects logistic regression, multiple linear regression, generalised estimating equations, Chi square test, scatter plot |
| **11** | Migot-Nabias F et al | Immune responses against Plasmodium falciparum asexual blood-stage antigens and disease susceptibility in Gabonese and Cameroonian children | 1999 | Incidence of malaria episodes, parasitemia | 13 months | 186 | School children | Gabon, Cameroon | Descriptives, histogram, Fisher exact test, Chi square test, Mann-Whitney U test, log transformation, student t test, logistic regression |
| **12** | Witworth J et al | Effect of HIV-1 and increased immunosuppresion on malaria parasitemia and clinical episodes in adults in rural Uganda: a cohort study | 2000 | Clinical malaria, parasitaemia, or parasite density | 8 years | 484 | Adult population aged ≥13 years, HIV-1-seropositive | Uganda | Descriptives, logistic, simple linear regression, natural logarithm transformation, Cox regression, rate ratios, Trend test, Wald test |
| **13** | Conway DJ et al | A principle target of human immunity to malaria identified by molecular population genetic and immunological analyses | 2000 | Clinical malaria incidence | 5 months | 337 | Children 3–7 years of age | Gambia | Descriptives, sensitivity, specificity, positive predictive value, negative predictive value, Fisher's exact test, Chi square test, logistic regression |
| **14** | Dodoo D et al | Naturally acquired antibodies to the glutamate-rich protein are associated with protection against plasmodium falciparum malaria | 2000 | Incidence of clinical malaria episodes | 26 months | 115 | Children 3-15 years negative for sickle-cell trait | Ghana | Descriptives, Wilcoxon signed-rank test, Mann-Whitney rank sum test, spearman's rank order correlation coefficient, multiple logistic regression |
| **15** | Riley EM et al | Lack of Association between Maternal Antibody and Protection of African Infants from Malaria Infection | 2000 | Malaria parasitemia infection | 20 weeks | 143 | infants born to mothers recruited in the last trimester of pregnancy | Ghana | Descriptives, t test, Poisson regression, simple linear regression, Cox regression |
| **16** | Migot-Nabias F et al | Immune response to Plasmodium falciparum liver stage antigen-1: geographical variations within Central Africa and their relationship with protection from clinical malaria | 2000 | Prevalence of malaria, parasitemia | 1 year | 76 | School children | Gabon, Cameroon | Descriptives, Chi square test, student t test, Mann-Whitney U test, log transformation, univariate logistic regression |
| **17** | Diagne N et al | Increased susceptibility to malaria during the early postpartum period | 2000 | Incidence of malaria episodes, parasitemia | 33 months | 38 | pregnant women | Senegal | Descriptives, generalised estimating equations, Wald test |
| **18** | Kun JF et al | Nitric oxide synthase, increased nitric oxide production, and protection against malaria | 2001 | Rates of malarial attacks, time to first malaria reinfection | 29 months | 200 | Children suffering severe malaria matched by age, sex and provenance with children with mild malaria | Gabon | Descriptives, Mann-Whitney U test, Wilcoxon rank-sum test, Breslow test, Kaplan Meier estimator |
| **19** | Franks S et al | Frequent and persistent, asymptomatic Plasmodium falciparum infections in African infants, characterized by multilocus genotyping | 2001 | Recurrent episodes of malaria infection | 2 years | 143 | New-born infants | Ghana | Descriptives, histograms |
| **20** | May J et al | HLA-DQB1*0501-Restricted Th1 Type Immune Responses to Plasmodium falciparum Liver Stage Antigen 1 Protect against Malaria Anemia and Reinfections | 2001 | Incidence of malarial reinfections | 52 months | 179 | Children and adults, above 4 years | Gabon | Descriptives, contingency table, analyses, Chi square test, Mann-Whitney U test, Kaplan Meier estimator, log rank test |
| **21** | Ekvall H et al | Hemoglobin concentration in children in a malaria holoendemic area is determined by cumulated Plasmodium falciparum parasite densities | 2001 | Incidence of malaria episodes, parasitemia | 5 months | 211 | Children < 24 months of age | Tanzania | Descriptives, Chi square test, Fisher exact test, student t test, log transformation, Wilcoxon rank-sum test, simple linear regression |
| **22** | Baird JK et al | Seasonal malaria attack rates in infants and young children in northern Ghana | 2002 | Incidence density of parasitemia and malaria disease | 31 months | 536 | Children between the ages of six and 24 months | Ghana | Descriptives, Fisher exact test, Chi square test, life table method |
| **23** | Zhou A et al | Prevalence of Plasmodium falciparum infection in pregnant Cameroonian women | 2002 | Prevalence of Plasmodium falciparum infection | 9 months | 719 | pregnant women attending antenatal clinic | Cameroon | Descriptives, t test, Fisher exact test, Chi square test, logistic generalised estimating equations, log transformation |
| **24** | Domarle O et al | Family analysis of malaria infection in Dienga, Gabon | 2002 | Changes in levels of parasitemia | 8 months | 50 | Children, 1-17 years | Gabon | Descriptives, Kruskal-Wallis test, Mann-Whitney U test, Welch test, Scheff test, Chi square test, arithmetic mean, correlation scatter plot, time series |
| **25** | Dorsey G et al | Sulfadoxine/pyrimethamine alone or with amodiaquine or artesunate for treatment of uncomplicated malaria: a longitudinal randomised trial | 2002 | Repeated malaria episodes, parasitemia | 1 year | 316 | healthy children aged 6 months to 5 years | Uganda | Descriptives, negative binomial regression, log transformation, Chi square or Fisher exact test, Kaplan Meier estimator, log rank test, generalised estimating equations with robust standard errors |
| **26** | Ladner J et al | HIV infection, malaria, and pregnancy: a prospective cohort study in Kigali, Rwanda | 2002 | Incidence of clinical malaria, time to first episode | 9 months | 457 | HIV-positive and HIV-negative pregnant women | Rwanda | Descriptives, student t test, Chi square test, log rank test, incidence density rate, Kaplan Meier, relative risks, odds ratios, Cox regression |
| **27** | Staedke SG et al | Proximity to mosquito breeding sites as a risk factor for clinical malaria episodes in an urban of Ugandan children | 2003 | Incidence of clinical malaria | 12 months | 305 | Healthy children between the ages of 6 and 59 months | Uganda | Descriptives, multiple negative binomial regression, time series |
| **28** | Spiengel A et al | Increased frequency of malaria attacks in subjects co-infected by intestinal worms and Plasmodium falciparum malaria | 2003 | Incidence of clinical malaria attacks | 1 year | 80 | Children aged 1-14 years | Senegal | Descriptives, multiple Poisson regression |
| **29** | Metzger WG | Serum IgG3 to the Plasmodium falciparummerozoite surface protein 2 is strongly associated with a reduced prospective risk of malaria | 2003 | Incidence of clinical malaria, parasitemia | 5 months | 329 | Children aged 3–7 years living in villages | Gambia | Descriptives, spearman rank correlation, Mann-Whitney U test, multiple logistic regression, Chi square, relative risks, binomial regression with a log-link |
| **30** | Cattamanchi A et al | Distinguishing recrudescence from reinfection in a longitudinal antimalarial drug efficacy study: comparison of results based on genotyping of msp-1, msp-2, and glurp | 2003 | Incidence of malaria episodes, time to re-infection | 12 months | 316 | Healthy children between the ages of 6 months and 5 enrolled from the community | Uganda | Descriptives, Cox regression, Kaplan Meier estimator, log rank test, histogram |
| **31** | Njama D et al | Urban malaria: primary caregivers' knowledge, attitudes, practices and predictors of malaria incidence in a cohort of Ugandan children | 2003 | Incidence of malaria episodes | 2 years | 307 | Children aged 6 months to 5 years | Uganda | Descriptives, Chi square test, student t test, multiple negative binomial regression, histogram |
| **32** | Duarte EC et al | Self-reported compliance with last malaria treatment and occurrence of malaria during follow-up in a Brazilian Amazon population | 2003 | Incidence of malaria episodes, parasitemia | 8 months | 414 | Children and adults in community | Brazil | Descriptives, simple linear regression, multiple linear regression, Cox regression, Fisher exact test, Chi square test, log rank test |
| **33** | Flanagan KL et al | Ex vivo interferon-gamma immune response to thrombospondin-related adhesive protein in coastal Kenyans: longevity and risk of Plasmodium falciparum infection | 2003 | Incidence of malaria episodes, time to the first parasitemia | 1 year | 217 | Children and adults between 1 month and 81 years | Kenya | Descriptives, Fisher exact test, student t test, Kaplan Meier estimator, Cox regression, log rank test |
| **34** | Njama-meya D et al | Asymptotic parasitaemia as a risk factor for symptomatic malaria in a cohort of Ugandan children | 2004 | Incidence of asymptomatic parasitaemia, symptomatic malaria | 24 months | 283 | Children aged 6 months to 5 years recruited from the community | Uganda | Descriptives, generalised estimating equations with exchangeable correlation and robust standard errors, life table analysis, natural log transformation, multiple logistic regression, Kaplan Meier product limit formula |
| **35** | Cavanagh DR et al | Antibodies to the N-terminal block 2 of Plasmodium falciparum merozoite surface protein 1 are associated with protection against clinical malaria | 2004 | Incidence of clinical malaria episodes, time to first clinical malaria episode | 17 months | 280 | Children aged 3 to 15 years, typed negative for sickle cell trait | Ghana | Chi square test, Fisher exact test, multiple logistic regression, Cox regression, Kaplan Meier estimator |
| **36** | Pascal R et al | Hemoglobin C is associated with reduced Plasmodium falciparum parasitemia and low risk of mild malaria attack | 2004 | Incidence of parasitemia and mild malaria attacks | 2 years | 256 | Health children and adults | Burkina Faso | Descriptives, Wald test, Chi square test, logistic regression, student's t test, Monte Carlo test, simple linear regression, logarithmic transformation |
| **37** | Meraldi V et al | Natural antibody response to Plasmodium falciparum Exp-1, MSP-3 and GLURP long synthetic peptides and association with protection | 2004 | Incidence of clinical malaria episodes, parasitemia | 7 months | 293 | Children aged 6 months to 9 year | Burkina Faso | Descriptives, Wilcoxon signed-rank test, Mann-Whitney U test, rank-sum test, Chi square, multiple logistic regression, Kaplan Meier estimator |
| **38** | Henning L et al | A prospective study of Plasmodium falciparum multiplicity of infection and morbidity in Tanzanian children | 2004 | Incidence of malaria episodes, parasitemia, time to first episode | 9 months | 610 | Children aged 0-6 years | Tanzania | Descriptives, Kaplan Meier estimator, Chi square test, log rank test |
| **39** | Erhart A et al | Forest malaria in Vietnam: a challenge for control | 2004 | Incidence of malaria episodes, parasitemia | 2 years | 573 | Children and adults | Vietnam | Descriptives, incidence rates, generalised estimating equations, time series plot |
| **40** | Mwangi TW et al | Case definitions of clinical malaria under different transmission conditions in Kilifi district, Kenya | 2005 | Incidence of clinical malaria episodes, parasite density | 30 months | 1602 | Children and adults from randomly selected households in mapped clusters | Kenya | Descriptives, logistic regression, Poisson regression |
| **41** | Patnaik P et al | Effects of HIV-1 serostatus, HIV-1 RNA concentration, and cd4 cell count on the incidence of malaria infection in a cohort of adults in rural Malawi | 2005 | Incidence of malaria parasitemia | 9 months | 349 | Adults with known HIV-1 serostatus who were aparasitaemic | Malawi | Descriptives, multiple Poisson regression with robust standard errors, Cox regression, conditional risk set models for ordered events |
| **42** | Lyke KE et al | Association of Schistosoma haematobium infection with protection against acute Plasmodium falciparum malaria in Malian children | 2005 | Time to the first clinical malaria infection, incidence of malaria episodes, and parasitemia | 25 weeks | 676 | Children 4-14 years of age with and without asymptomatic S. haematobium infection | Mali | Descriptives, Wilcoxon signed-rank test, student t test, Chi square test, Fisher exact test |
| **43** | Bach O et al | Falciparum malaria after splenectomy: a prospective controlled study of 33 previously splenectomized Malawian adults | 2005 | Incidence and severity of malarial infections and illness | 14 months | 66 | Splenectomized adults and controls | Malawi | Descriptives, student t test, Fisher exact test, Mann-Whitney U test, Kolmogorov Smirnov test |
| **44** | Mermin J et al | Effect of co-trimoxazole prophylaxis, antiretroviral therapy, and insecticide-treated bed nets on the frequency of malaria in HIV-1-infected adults in Uganda: a prospective cohort study | 2006 | Incidence of clinical malaria, parasite density | 5 months | 1035 | HIV-infected individuals aged 18 years or older | Uganda | Descriptives, Poisson regression with a log-link, generalised estimating equations with exchangeable correlation structure, log-transformation, geometric means |
| **45** | Laufer MK et al | Impact of HV-associated immunosuppression on malaria infection and disease in Malawi | 2006 | Incidence of clinical malaria episodes, parasite density | 23 months | 660 | Adults >16 years living with HIV infection | Malawi | Descriptives, Poisson goodness of fit to test data overdispersion, Huber white sandwich estimator, Chi square test, simple linear regression, logistic regression, Poisson regression, incidence rates |
| **46** | Polley SD et al | High levels of serum antibodies to merozoite surface protein 2 of Plasmodium falciparum are associated with reduced risk of clinical malaria in coastal Kenya | 2006 | Incidence of malaria episodes, parasitemia | 28 weeks | 170 | Children and adults aged 3 weeks to 85 years | Kenya | Descriptives, multiple logistic regression, Wilcoxon rank-sum test, Chi square test for trend |
| **47** | Touré FS et al | Analysis of human antibodies to erythrocyte binding antigen 175 peptide 4 of Plasmodium falciparum | 2006 | Incidence of malaria episodes, parasitemia | 13 months | 158 | School children | Gabon | Descriptives, histograms, Chi square test, Mann-Whitney U test, log transformation, student t test, logistic regression |
| **48** | Sarr JB et al | Impact of red blood cell polymorphisms on the antibody response to Plasmodium falciparum in Senegal | 2006 | Incidence of malaria episodes, parasitemia | 18 months | 413 | Children aged from 2 to 10 years | Senegal | Descriptives, Chi square test, student t test, Mann-Whitney U test, Kruskal-Wallis, multiple linear regression |
| **49** | Malamba SS et al | Effect of cotrimoxazole prophylaxis taken by human immunodeficiency virus (HIV)-infected persons on the selection of sulfadoxine-pyrimethamine-resistant malaria parasites among HIV-uninfected household members | 2006 | Incidence of malaria episodes, parasitemia | 1 year | 2567 | HIV-uninfected household members | Uganda | Descriptives, multiple Poisson regression, Chi square test, generalised estimating equations |
| **50** | Michon P et al | The Risk of Malarial Infections and Disease in Papua New Guinean children | 2007 | Incidence of clinical malaria episodes, parasitemia | 25 weeks | 206 | School children aged 5–14 years | Papua New Guinea | Descriptives, Cox regression, log rank test, Poisson regression, likelihood ratio tests, Kaplan Meier curves |
| **51** | Bousema JT et al | A longitudinal study of immune responses to Plasmodium falciparum sexual stage antigens in Tanzanian adults | 2007 | Prevalence of parasitemia and immune reactivity | 16 months | 43 | Children and adults 16 years of age or older in community | Tanzania | Descriptives, natural log transformation, Wilcoxon rank-sum test, Pearson correlation coefficient, multiple logistic regression with generalised estimating equations |
| **52** | Dicko A et al | Year-to-year variation in the age-specific incidence of clinical malaria in two potential vaccine testing sites in Mali with different levels of malaria transmission intensity. | 2007 | Incidence of malaria episodes, parasitemia | 1 year | 795 | Children 3 months to 2 years of age | Mali | Descriptives, Poisson generalised estimating equations, Fisher exact test, Chi square test, time series plot |
| **53** | Kamya MR et al | Effects of trimethoprim-sulfamethoxazole and insecticide-treated bed nets on malaria among HIV-infected Ugandan children | 2007 | Incidence of malaria episodes, parasitemia | 11 months | 861 | HIV-infected and health community-based children | Uganda | Descriptives, log transformation, Chi square test, Fisher exact test, Wilcoxon rank-sum test, student t-test, negative binomial regression |
| **54** | Clark TD et al | Factors determining the heterogeneity of malaria incidence in children in Kampala, Uganda | 2008 | Incidence of malaria episodes | 24 months | 558 | Children aged 1-10 years recruited from a census population | Uganda | Descriptives, generalised estimating equations, Kulldorff's spatial scan statistic, multiple Poisson regression |
| **55** | D'ombrain et al | Association of early interferon-y production with immunity to clinical malaria: a longitudinal stud among Papua New Guinean children | 2008 | Incidence of clinical malaria | 6 months | 206 | School-aged children | Papua New Guinean | Descriptives, log transformation, geometric means compared with student's t test, Poisson regression, Cox regression, spearman's rank correlation |
| **56** | Machault V et al | Remote Sensing and Malaria Risk for Military Personnel in Africa | 2008 | Incidence rate of clinical malaria attacks | 8 months | 1189 | French military personnel in Africa | Côte d’Ivoire, Central African Republic, Chad, Senegal, Republic of Djibouti | Descriptives, multiple Poisson regression, random mixed-effects regression |
| **57** | Crompton PD et al | Sickle cell trait is associated with a delayed onset of malaria: implications for time to event analysis in clinical studies of malaria | 2008 | Incidence of malaria episodes, time to malaria episodes | 8 months | 225 | Children and adults aged 2-25 years | Mali | Descriptives, Fisher exact, Kruskal-Wallis tests, Kaplan Meier estimator, log rank test, Cox regression, multiple Poisson regression |
| **58** | da Silva-Nunes M et al | Malaria on the Amazonian frontier: transmission dynamics, risk factors, spatial distribution, and prospects for control | 2008 | Incidence of symptomatic malaria episodes | 14.5 months | 509 | Children and adults m 1 month to 90 years of age in community | Brazil | Descriptives, mixed-effects logistic regression, stratification, likelihood ratio test, multiple, logistic additive regression |
| **59** | Nkuo-Akenji T et al | High prevalence of HIV and malaria co-infection in urban Douala, Cameroon | 2008 | Prevalence and incidence of malaria, parasitemia | 6 months | 684 | Adult hospital attendees | Cameroon | Descriptives, student t-test, ANOVA test, Chi square test |
| **60** | Schwarz NG et al | Placental malaria increases malaria risk in the first 30 months of life | 2008 | Incidence of malaria, parasitemia, time to first episode of malaria | 30 months | 527 | New-borns | Gabon | Descriptives, Cox regression, Chi square test, student t test, Wilcoxon rank-sum test, log rank test, Kaplan Meier estimator |
| **61** | John CC et al | Antibodies to pre-erythrocytic Plasmodium falciparum antigens and risk of clinical malaria in Kenyan children | 2008 | Incidence of malaria episodes, times to development of Plasmodium falciparum parasitemia | 52 weeks | 86 | Children aged >3 months and <8 years | Kenya | Descriptives, Chi square test, spearman correlation, multiple linear regression, Cox regression, Kaplan Meier estimator, negative binomial regression, log rank test |
| **62** | Ogutu BR et al | Blood stage malaria vaccine eliciting high antigen-specific antibody concentrations confers no protection to young children in western Kenya | 2009 | Time to first clinical episodes of Plasmodium falciparum malaria, average number of clinical episodes | 25 months | 400 | Children aged 12-47 months in general good health | Kenya | Descriptives, Fisher exact, log rank, Cox regression, Kaplan Meier estimator, multiple Poisson regression |
| **63** | Arinaitwe E et al | Artemether-lumefantrine versus dihydroartemisinic-piperaquine for falciparum malaria: a longitudinal, randomized trial in young Ugandan children | 2009 | Incidence of clinical malaria episodes, recurrent falciparum parasitemia | 27 months | 351 | Children who were at least 4 months of age, weighted at least 5 kg, and diagnosed with first episode of uncomplicated malaria | Uganda | Descriptives, Kaplan Meier, Cox regression, negative binomial regression |
| **64** | Malhotra I et al | Can prenatal malaria exposure produce an immune tolerant phenotype? a prospective birth cohort study in Kenya | 2009 | Incidence of malaria episodes | 3 years | 586 | New-borns to mothers recruited from antenatal clinic | Kenya | Descriptives, Chi square test, Mann-Whitney U test, Kruskal-Wallis test, student t test, ANOVA test, binomial generalised estimating equations, mixed-effects regression, log transformation |
| **65** | Greenhouse B et al | Decreasing efficacy of antimalarial combination therapy in Uganda is explained by decreasing host immunity rather than increasing drug resistance | 2009 | Incidence of uncomplicated malaria episodes, asymptomatic parasitemia | 29 months | 129 | Children aged 1–10 years selected randomly from community | Uganda | Descriptives, Kaplan Meier product limit formula, Cox regression, multiple logistic regression, robust inference with jack-knife method |
| **66** | Jensen TP et al | Use of the slide positivity rate to estimate changes in malaria incidence in a cohort of Ugandan children | 2009 | Incidence of malaria episodes, parasitemia | 4 years | 690 | Children aged 1-10 years | Uganda | Descriptives, student t test, ANOVA test, negative binomial regression |
| **67** | Robinson LJ et al | Cellular tumour necrosis factor, gamma interferon, and interleukin-6 responses as correlates of immunity and risk of clinical Plasmodium falciparum malaria in children from Papua New Guinea | 2009 | Incidence of malaria episodes, parasitemia | 6 months | 165 | Children of 5 to 14 years | Papua New Guinea | Descriptives, Chi square test, student t test, log transformation, likelihood ratio test, multiple Poisson regression, Cox regression |
| **68** | Neuberger et al | A Cohort study of risk factors for malaria among health care workers in Equatorial Guinea: stay away from the ground floor | 2010 | Time to malaria, incidence of malaria episodes | 15 months | 102 | Healthcare workers in a hospital | Equatorial Guinea | Descriptives, univariate analysis of incidence rate ratio, Fisher exact test, Kaplan Meier, Cox regression, log rank, maximum likelihood ratio test |
| **69** | Leke RFG et al | Longitudinal Studies of Plasmodium falciparum Malaria in Pregnant Women Living in a Rural Cameroonian Village with High Perennial Transmission | 2010 | Incidence of clinical malaria, parasitemia | 9 months | 60 | Pregnant women attending antenatal clinic | Cameroon | Descriptives, Chi square test, Wilcoxon rank-sum test, likelihood ratio test, generalised estimating equations, multiple Poisson regression |
| **70** | Lin E et al | Differential Patterns of Infection and Disease with P. falciparum and P. vivax in Young Papua New Guinean Children | 2010 | Prevalence of parasitemia, incidence of clinical episodes | 16 months | 264 | Children 1–3 years of age | Papua New Guinea | Descriptives, Wald’s Chi square tests, normal generalised estimating equations |
| **71** | Baliraine FN et al | A cohort study of Plasmodium falciparum infection dynamics in Western Kenya Highlands | 2010 | Incidence of clinical malaria episodes, infection duration | 1 year | 246 | School children 5-17 years randomly selected from community | Kenya | Descriptives, Fisher exact test, paired t test, Wilcoxon signed-rank test, analysis of molecular variance (AMOVA), Kaplan Meier, Tukey Kramer test |
| **72** | Nahum A et al | Malaria incidence and prevalence among children living in a peri-urban area on the coast of Benin, west Africa: a longitudinal study | 2010 | Incidence and prevalence of clinical malaria episodes | 18 months | 553 | Children 6–59 months with no chronic illness | Benin | descriptive statistics, Wald tests, multiple logistic regression with robust standard errors |
| **73** | Akpogheneta OJ et al | Boosting antibody responses to Plasmodium falciparum merozoite antigens in children with highly seasonal exposure to infection | 2010 | Incidence of clinical malaria episodes, parasitemia | 10 months | 103 | Children under 74 months of age | Gambia | Descriptives, two sample t test, Wilcoxon rank-sum test, linear regression, unadjusted Pearson’s coefficient |
| **74** | Crompton PD et al | In vitro growth-inhibitory activity and malaria risk in a cohort study in Mali | 2010 | Incidence of clinical malaria episodes, parasitemia | 9 months | 225 | Children aged 2 to 10 years and adults 18 to 25 years, with no anemia, fever, acute systemic illness or pregnancy | Mali | Descriptives, Kruskal-Wallis test, Fisher exact test, Kaplan Meier estimator, log rank test, logistic regression Hosmer and Lemeshow goodness-of-fit test, Cox regression, Poisson regression with bootstrap test and confidence intervals, and the deviance goodness-of-fit test |
| **75** | Clark TD et al | Incidence of malaria and efficacy of combination antimalarial therapies over 4 years in an urban cohort of Ugandan children | 2010 | Recurrent malaria episodes, parasitemia | 2 years | 690 | Children aged 1-10 years were enrolled from randomly selected households | Uganda | Descriptives, Kaplan Meier product limit formula, time series plot, generalised estimating equations |
| **76** | Weiss GE et al | The Plasmodium falciparum-specific human memory B cell compartment expands gradually with repeated malaria infections | 2010 | Incidence of malaria episodes, parasitemia | 1 year | 185 | Children and adults aged 2 to 25 years | Mali | Descriptives, Kaplan Meier estimator, Kruskal-Wallis, Fisher exact test, Wilcoxon signed-rank test, spearman correlation coefficient, Cox regression, log rank test, logistic, Poisson regression |
| **77** | Jaenisch T et al | Malaria incidence and prevalence on Pemba island before the onset of the successful control intervention on the Zanzibar archipelago | 2010 | Incidence of malaria episodes, parasitemia | 6 months | 537 | Children aged 1-23 months | Tanzania | Descriptives, time series plots, incidence rates |
| **78** | Roussilhon C et al | Understanding human-Plasmodium falciparum immune interactions uncovers the immunological role of worms | 2010 | Incidence of malaria episodes, parasitemia | 51 months | 203 | Children 1–14 years of age | Senegal | Descriptives, nominal logistic regression, log transformation, multiple linear regression |
| **79** | Vora N et al | Breastfeeding and the risk of malaria in children born to HIV-infected and uninfected mothers in rural Uganda | 2010 | Incidence of malaria episodes, parasitemia | 2 years | 346 | HIV-unexposed, HIV-exposed and HIV-infected children | Uganda | descriptives, sample size, Mann-Whitney U test, Chi square test, stratification, binomial generalised estimating equations |
| **80** | Thévenon AD et al | Elevated levels of soluble TNF receptors 1 and 2 correlate with Plasmodium falciparum parasitemia in pregnant women: potential markers for malaria-associated inflammation | 2010 | Incidence of malaria, parasitemia | 12 months | 282 | Pregnant women | Cameroon | Descriptives, spearman correlation coefficient, chi square test, Mann-Whitney U test, Wilcoxon rank-sum test, likelihood ratio test, mixed-effects regression, multiple linear regression |
| **81** | Richards JS et al | Association between naturally acquired antibodies to erythrocyte-binding antigens of Plasmodium falciparum and protection from malaria and high-density parasitemia | 2010 | Incidence of malaria episodes, time to first re-infection or first symptomatic episode | 6 months | 206 | Children aged 5–14 years | Papua New Guinean | Descriptives, Wilcoxon rank-sum test, Kruskal-Wallis test, Kaplan Meier estimator, spearman rank test, log rank test, Cox regression |
| **82** | Sowunmi A et al | Factors contributing to delay in parasite clearance in uncomplicated falciparum malaria in children | 2010 | Incidence of parasitemia, delay in parasite clearance | 42 days | 2752 | Children with symptoms of acute falciparum malaria aged 144 months or below | Nigeria | Descriptives, student t test, Chi square test, ANOVA test, Mann-Whitney test, Kruskal-Wallis test, Wilcoxon rank-sum test, multiple logistic regression |
| **83** | Giha HA et al | Association of a single nucleotide polymorphism in the C-reactive protein gene (-286) with susceptibility to Plasmodium falciparum malaria | 2010 | Incidence of malaria episodes, parasitemia | 9 years | 192 | Health children and adults | Sudan | Descriptives, Pearson correlation, Kruskal-Wallis, one-way ANOVA test, Chi square test, Fisher exact test |
| **84** | Loucoubar C et al | Impact of Changing Drug Treatment and Malaria Endemicity on the Heritability of Malaria Phenotypes in a Longitudinal Family-Based Cohort Study | 2011 | Number of clinical malaria episodes | 19 years | 210 | Children and adults who were volunteers from community | Senegal | Descriptives, scatter plot, time series, histogram, variance component analysis, generalized linear mixed-effects- multiple regression |
| **85** | Huynh BT et al | Malaria associated symptoms in pregnant women followed-up in Benin | 2011 | Incidence of malaria infection and disease | 6 months | 982 | Pregnant women with gestational age under 24 weeks | Benin | Descriptives, multiple logistic regression, Chi square or Fisher exact test, student t test |
| **86** | Dodoo D et al | Antibody levels to multiple malaria vaccine candidate antigens in relation to clinical malaria episodes in children in the Kasena-Nankana district of Northern Ghana | 2011 | Incidence of clinical malaria episodes | 1 year | 325 | Children aged 1 to 6 years | Ghana | Descriptives, negative binomial regression |
| **87** | Manning L et al | Features and Prognosis of Severe Malaria Caused by Plasmodium falciparum, Plasmodium vivax and Mixed Plasmodium Species in Papua New Guinean Children | 2011 | Incidence of malaria episodes, parasitemia | 3 years | 340 | Children aged 0.5–10 years with severe malaria | Papua New Guinea | Descriptives, multiple logistic regression, Bonferroni correction, Dunn test, contingency table, Fisher exact test |
| **88** | Yamazaki A et al | Human leukocyte antigen class I polymorphisms influence the mild clinical manifestation of Plasmodium falciparum infection in Ghanaian children | 2011 | Incidence of malaria episodes, parasitemia | 12 months | 429 | Healthy children of 3–11 years of age | Ghana | Descriptives, attributable risk, relative risk, Bonferroni test, ANOVA test, student t test, multiple logistic regression |
| **89** | Nakanjako D et al | Low prevalence of Plasmodium falciparum antigenemia among asymptomatic HAART-treated adults in an urban cohort in Uganda | 2011 | Changes in parasitemia levels | 6 months | 128 | Adults treated with asymptomatic HAART | Uganda | Descriptives |
| **90** | Cech PG et al | Virosome-formulated Plasmodium falciparum AMA-1 & CSP derived peptides as malaria vaccine: randomized phase 1b trial in semi-immune adults & children. | 2011 | incidence of malaria episodes, parasitemia | 365 days | 50 | Children and adults | Tanzania | Descriptives, sample size, Fisher exact test, Wilcoxon rank-sum test, Kaplan Meier estimator, log rank test, Cox regression, multiple Poisson regression |
| **91** | Ouwe-Missi-Oukem-Boyer O et al | Hepatitis C Virus Infection May Lead to Slower Emergence of P. falciparum in Blood | 2011 | Incidence of malaria, parasitemia, time to first episode of malaria | 1 year | 319 | Children and adults | Gabon | Descriptives, student t test, Chi square test, Trend test, Cox regression, Kaplan Meier estimator, log rank test |
| **92** | Sutcliffe CG et al | Reduced risk of malaria parasitemia following household screening and treatment: A cross-section and longitudinal cohort study | 2012 | frequency of Rapid diagnostic test positivity | 1 year | 390 | Healthy children and adults enrolled from households | Zambia | Descriptives, logistic regression with random intercept mixed-effects, Poisson regression with robust std errors |
| **93** | Mueller I et al | Force of infection is key to understand the epidemiology of plasmodium falciparum malaria in Papua New Guinean children | 2012 | Number of clinical malaria episodes | 16 months | 264 | Children 1–3 years of age | Papua New Guinea | Descriptives, generalised linear mixed-effects regression |
| **94** | Jagannathan P et al | Increasing incidence of malaria in children despite insecticide-treated bed nets and prompt anti-malarial therapy in Tororo, Uganda | 2012 | Incidence of malaria and prevalence of asymptomatic parasitaemia | 48 months | 100 | Children aged six weeks to 10 months | Uganda | Descriptives, generalised estimating equations with robust standard errors, Generalized additive regression with smoothing splines |
| **95** | Loha E et al | Predictors of Plasmodium falciparum malaria incidence in Chano Mille, South Ethiopia: a longitudinal study. | 2012 | Plasmodium falciparum malaria incidence | 101 weeks | 8121 | All residents in a community | Ethiopia | Descriptives, time series, Ljung-Box Q statistics was used as regression diagnostics, multiple Poisson regression, omnibus test, generalised estimating equations, Chi square test |
| **96** | Laufer MK et al | A longitudinal trial comparing chloroquine as monotherapy or in combination with artesunate, azithromycin or atovaquone-proguanil to treat malaria | 2012 | Incidence of clinical malaria | 1 year | 640 | Children with uncomplicated malaria | Malawi | Descriptives, Poisson regression, Kaplan Meier estimator, Cox regression, Kruskal-Wallis test, generalised estimating equations |
| **97** | Zhang G et al | Interleukin-10 (IL-10) polymorphisms are associated with IL-10 production and clinical malaria in young children | 2012 | Incidence of clinical malaria episodes | 24 months | 240 | Infants born to HIV-negative mothers | Mozambique | Descriptives, principal component analysis, ANOVA test, independent sample t test, multiple linear regression, Poisson regression, log transformation |
| **98** | Kobayash T et al | Temporal and spatial patterns of serologic responses to Plasmodium falciparum antigens in a region of declining malaria transmission in southern Zambia | 2012 | Incidence of malaria episodes, parasitemia | 1 year | 442 | Children and adults in community | Zambia | Descriptives, linear mixed-effects with robust standard errors |
| **99** | Olotu A et al | Estimating individual exposure to malaria using local prevalence of malaria infection in the field | 2012 | Incidence of asymptomatic parasitaemia or malaria episodes | 12 years | 2425 | Children and adults in community | Kenya | Descriptives, ROC curve for the multivariable weighted local prevalence-based regressions, multiple logistic regression, modified Poisson regression, mixed-effects regression |
| **100** | Ezeamama AE et al | HIV infection and the incidence of malaria among HIV-exposed children from Tanzania | 2012 | Incidence of repeated malaria episodes | 2 years | 2320 | New-born infants of HIV-infected mothers | Tanzania | Descriptives, Kaplan Meier estimator, Andersen-Gill Cox regression, generalised estimating equations, likelihood ratio test |
| **101** | Dolo H et al | Filariasis attenuates anemia and proinflammatory responses associated with clinical malaria: a matched prospective study in children and young adults | 2012 | Incidence of clinical malaria, time to first episode, clinical signs and symptoms, and malaria parasitemia | 6 months | 62 | Children and young adults aged from 1 to 20 years | Mali | Descriptives, Poisson regression, Kaplan Meier estimator, Cox regression, Mann-Witney U test, Fisher exact test, log transformation, student t test, Wilcoxon signed-rank test, spearman rank test |
| **102** | Kynast-Wolf G et al | ITN protection, MSP1 antibody levels and malaria episodes in young children of rural Burkina Faso. | 2012 | Incidence of malaria episodes, parasitemia | 10 months | 120 | Infants recruited from community | Burkina Faso | Descriptives, log transformation, multiple regression, Wilcoxon signed-rank test, student t test |
| **103** | Burns M et al | Insecticide-treated plastic sheeting for emergency malaria prevention and shelter among displaced populations: an observational cohort study in a refugee setting in Sierra Leone | 2012 | Incidence of malaria episodes, time to first symptomatic or asymptomatic infection | 8 months | 89 | Children 4–36 months of age in refugee camp | Sierra Leone | Descriptives, mixed-effects regression, incidence rate ratio, Kaplan Meier estimator, Chi square test, log rank test, |
| **104** | Jonker FA et al | Iron status predicts malaria risk in Malawian preschool children | 2012 | Incidence of malaria episodes, parasitemia | 1 year | 727 | Preschool children | Malawi | Descriptives, marginal structural models, Cox regression, Wald test, sensitivity analysis |
| **105** | Valea I et al | An analysis of timing and frequency of malaria infection during pregnancy in relation to the risk of low birth weight, anaemia and perinatal mortality in Burkina Faso | 2012 | Incidence of malaria, parasitemia, time to re-infection | 2 years | 1034 | Pregnant women | Burkina Faso | Descriptives, student t test, chi square test, Fisher exact, Mann-Whitney U test, multiple Poisson regression with robust standard errors |
| **106** | Bollo MA et al | Sickle cell trait protects against Plasmodium falciparum infection | 2012 | Incidence of malaria parasitemia | 2 years | 621 | Children recruited from community | Mali | Descriptives, student t test, multiple multinomial logistic regression, log transformation, Kaplan Meier estimator, log rank test, Cox regression, Chi square test |
| **107** | Minja DT et al | Reliability of rapid diagnostic tests in diagnosing pregnancy-associated malaria in north-eastern Tanzania | 2012 | Incidence of malaria episodes, parasitemia | 9 months | 924 | Pregnant women | Tanzania | Descriptives, sensitivity, specificity, positive predictive value, negative predictive value |
| **108** | Arinaitwe E et al | The association between malnutrition and the incidence of malaria among young HIV-infected and -uninfected Ugandan children: a prospective study | 2012 | Incidence of malaria episodes | 2.5 years | 349 | HIV-infected and -uninfected children aged 6 weeks to 1 year | Uganda | Descriptives, generalised estimating equations with robust standard errors, negative binomial regression |
| **109** | Omar AH et al | Toll-like receptor 9 (TLR9) polymorphism associated with symptomatic malaria: a cohort study | 2012 | Incidence of symptomatic malaria, parasitemia | 1 year | 429 | Children aged 3-11 years | Ghana | Descriptives, Bonferroni correction, student t test, ANOVA test |
| **110** | Rosanas-Urgell A et al | Lack of associations of α(+)-thalassemia with the risk of Plasmodium falciparum and Plasmodium vivax infection and disease in a cohort of children aged 3-21 months from Papua New Guinea | 2012 | Incidence of malaria episodes, parasitemia | 21 months | 1112 | Children aged 3-21 months | Papua New Guinea | Descriptives, generalised estimating equations, Chi square test, multiple negative binomial regression |
| **111** | Kajeguka D et al | CD36 c.1264 T>G null mutation impairs acquisition of IgG antibodies to Plasmodium falciparum MSP1₁₉ antigen and is associated with higher malaria incidences in Tanzanian children | 2012 | Incidence of malaria episodes, parasitemia | 12 months | 711 | Children between 1 and 5 years | Tanzania | Descriptives, student t test, Chi square test, Fisher exact test, histogram |
| **112** | Abuaku B et al | Therapeutic efficacy of artemether-lumefantrine combination in the treatment of uncomplicated malaria among children under five years of age in three ecological zones in Ghana | 2012 | Change in parasitemia levels, time to parasitemia | 28 days | 175 | Children aged 6 months to 59 months presenting at the Out-Patient Department | Ghana | Descriptives, Fisher exact test, Chi square test, student t test, time series, log transformation |
| **113** | Tran TM et al | An intensive longitudinal cohort study of Malian children and adults reveals no evidence of acquired immunity to plasmodium falciparum. | 2013 | Incidence of clinical malaria episodes, Plasmodium falciparum infection | 7 months | 251 | Healthy children and adults aged 4-25 years who were free of blood-stage Plasmodium infection | Mali | Descriptives, Kaplan Meier, log rank test, Kruskal-Wallis test, Fisher exact test, Pearson correlation |
| **114** | Kalayjian BC et al | Marked Decline in Malaria Prevalence among Pregnant Women and Their Offspring from 1996 to 2010 on the South Kenyan Coast | 2013 | Incidence of malaria, parasitemia | 3 years | 953 | Children from birth to 3 years | Kenya | Descriptives, time series plots, cumulative percentage plots, histograms, cumulative risk of Plasmodium falciparum infection as a function of age is calculated over time using generalised estimating equations |
| **115** | Yewhalaw D et al | The effect of dams and seasons on malaria incidence and anopheles abundance in Ethiopia | 2013 | Incidence of clinical malaria episodes | 2 years | 2080 | Children less than 10 years randomly selected from community | Ethiopia | Descriptives, frailty model, mixed-effects Poisson regression |
| **116** | Getachew Y et al | Coping with time and space in modelling malaria incidence: a comparison of survival and count regression models | 2013 | Incidence of malaria | 23 months | 2082 | Children younger than 10 years living in villages | Ethiopia | Descriptives, mixed-effects Poisson, marginal hazard model, Cox regression, time series |
| **117** | Hill D et al | Opsonising Antibodies to P. falciparum Merozoites Associated with Immunity to Clinical Malaria | 2013 | Incidence of clinical malaria episodes, Plasmodium falciparum infections | 6 months | 198 | School children aged 5–14 years | Papua New Guinea | Descriptives, multivariate Poisson regression, time series plots, two-sample Wilcoxon rank-sum test or Kruskal-Wallis test, Bland Altman test, spearman correlation, Cox regression |
| **118** | Kakuru A et al | The Effects of ACT Treatment and TS Prophylaxis on Plasmodium falciparum Gametocytaemia in a Cohort of Young Ugandan Children | 2013 | Incidence of recurrent parasitemia, malaria episodes | 2 years | 351 | Children age 6 weeks to 12 months | Uganda | Descriptives, generalised estimating equations with robust standard errors, Kaplan Meier estimator, log rank test, Cox regression |
| **119** | Loucoubar C et al | High Number of Previous Plasmodium falciparum Clinical Episodes Increases Risk of Future Episodes in a Sub-Group of Individuals | 2013 | Incidence of clinical Plasmodium falciparum episodes | 19 years | 726 | Children and adults in community | Senegal | Descriptives, Fisher exact test, student’s t test, ANOVA test, Scheffe test, likelihood ratio test, generalized linear mixed-effects regression, multiple Poisson regression |
| **120** | Herrant M et al | Asthma and atopic dermatitis are associated with increased risk of clinical Plasmodium falciparum malaria | 2013 | Incidence of clinical Plasmodium falciparum malaria episodes, parasitemia | 15 years | 143 | Children aged 4 months to 14 years | Senegal | Descriptives, generalised linear mixed-effects regression |
| **121** | Asante KP, et al | Placental malaria and the risk of malaria in infants in a high malaria transmission area in Ghana: a prospective cohort study | 2013 | Incidence of episodes of malaria parasitemia or clinical malaria | 12 months | 1855 | Infants born to women with or without placental malaria | Ghana | Descriptives, principal component analysis, Kaplan Meier estimator, Cox regression with robust standard errors |
| **122** | De Beaudrap P et al | Impact of malaria during pregnancy on pregnancy outcomes in a Ugandan prospective cohort with intensive malaria screening and prompt treatment | 2013 | Incidence of malaria episodes, parasitemia | 9 months | 1218 | Pregnant women | Uganda | descripitives, mixed-effects Poisson regression, log transformation, multiple linear regression, logistic regression, log-linear regression |
| **123** | Ouédraogo A et al | Malaria Morbidity in High and Seasonal Malaria Transmission Area of Burkina Faso | 2013 | Incidence of malaria episodes | 1 year | 555 | Children aged 0-5 years randomly selected from households | Burkina Faso | Descriptives, Kaplan Meier estimator, logistic regression |
| **124** | Creek DJ et al | Pharmacokinetic predictors for recurrent malaria after dihydroartemisinic-piperaquine treatment of uncomplicated malaria in Ugandan infants | 2013 | Incidence of malaria, recurrent parasitemia | 5 months | 107 | Children aged 6–24 months with uncomplicated malaria | Uganda | Descriptives, Kaplan Meier product limit formula, Cox regression, scatterplot |
| **125** | Moussiliou A | High rates of parasite recrudescence following intermittent preventive treatment with sulphadoxine-pyrimethamine during pregnancy in Benin | 2013 | Incidence of malaria, parasite recrudescence | 9 months | 982 | Pregnant women | Benin | Descriptives, Fisher exact test, Chi square test, Kruskal-Wallis test, histogram |
| **126** | Haque U et al | Risk factors associated with clinical malaria episodes in Bangladesh: a longitudinal study | 2013 | Incidence of malaria episodes | 2 years | 7822 | Children and adults in community | Bangladeshi | Descriptives, multiple negative binomial regression, likelihood ratio test |
| **127** | Gupta V et al | The seroprevalence of Helicobacter pylori and its relationship to malaria in Ugandan children | 2013 | Incidence of malaria episodes, parasitemia | 2 years | 200 | Children, aged 1-10 years | Uganda | Descriptives, logistic regression, negative binomial regression, generalised estimating equations |
| **128** | Harrington WE et al | Intermittent preventive treatment in pregnant women is associated with increased risk of severe malaria in their offspring | 2013 | Time to repeated malaria episodes, parasitemia | 192 weeks | 820 | Infants | Tanzania | Descriptives, Cox regression, generalised estimating equations, Kaplan Meier estimator |
| **129** | Ndibazza J et al | Associations Between Maternal Helminth and Malaria Infections in Pregnancy and Clinical Malaria in the Offspring: A Birth Cohort in Entebbe, Uganda | 2013 | Incidence of malaria episodes, parasitemia | 5 years | 2289 | New-borns | Uganda | Descriptives, likelihood ratio test, Cox regression with robust standard errors, mixed-effects regression |
| **130** | Laurens MB et al | Extended safety, immunogenicity and efficacy of a blood-stage malaria vaccine in Malian children: 24-month follow-up of a randomized, double-blinded phase 2 trial | 2013 | Incidence of malaria episodes, time to first clinical malaria episode | 24 months | 400 | Children aged 1–6 years without acute and chronic illnesses | Mali | Descriptives, Cox regression, Poisson regression, Kaplan Meier estimator, Fisher exact test |
| **131** | Olotu A et al | Four-year efficacy of RTS, S/AS01E and its interaction with malaria exposure | 2013 | Incidence of malaria episodes, time to repeated malaria episodes | 4 years | 447 | Health children who were 5 to 17 months | Kenya, Tanzania | Descriptives, multiple negative binomial regression, Andersen-Gill Cox regression, Fisher exact test |
| **132** | Wanzira H et al | longitudinal outcomes in a cohort of Ugandan children randomized to artemether-lumefantrine versus dihydroartemisinic-piperaquine for the treatment of malaria | 2014 | Recurrent parasitemia, malaria episodes | 28 months | 312 | Children from a postnatal clinic | Uganda | Descriptives, generalised estimating equations, Kaplan Meier, log rank test, Cox regression, negative binomial regression |
| **133** | Arnold BF et al | Serological Measures of Malaria Transmission in Haiti: Comparison of Longitudinal and Cross-Sectional Methods | 2014 | Incidence of malaria episodes | 9 years | 142 | Children ages ≤11 years | Haiti | Descriptives, reversible catalytic model using maximum likelihood |
| **134** | Boudová S Laufer M et al | Pregnant women are a reservoir of malaria transmission in Blantyre, Malawi | 2014 | Incidence of placental malaria, parasitemia | 9 months | 448 | Pregnant women | Malawi | Descriptives, Fisher exact test, logistic regression |
| **135** | Andagalu B et al | Longitudinal study on Plasmodium falciparum gametocyte carriage following artemether-lumefantrine administration in a cohort of children aged 12–47 months living in Western Kenya, a high transmission area | 2014 | Prevalence of asymptomatic and symptomatic malaria, parasitemia | 1 year | 270 | Asymptomatic children aged 12–47 months | Kenya | Descriptives, ANOVA test, Kaplan Meier estimator, Cox regression, robust regression |
| **136** | Kaddumukasa M et al | Malariometric indices from Iganga, Uganda: baseline characterization in preparation of GMZ2 vaccine trial | 2014 | Incidence of malaria episodes | 6 months | 748 | Children aged 12 - 60 months | Uganda | Descriptives, time series plots, Chi square test, two-sample t test |
| **137** | Osier FH et al | Opsonic phagocytosis of Plasmodium falciparum merozoites: mechanism in human immunity and a correlate of protection against malaria | 2014 | Incidence of clinical malaria episodes | 6 months | 396 | Children and adults | Kenya | Descriptives, multiple modified Poisson regression, Cox regression, Chi square or Fisher exact test, Wilcoxon rank-sum test |
| **138** | Muhindo MK et al | Early parasite clearance following artemisinin-based combination therapy among Ugandan children with uncomplicated Plasmodium falciparum malaria | 2014 | Incidence of uncomplicated Plasmodium falciparum malaria, parasitemia episodes | 14 months | 202 | Children four to five years old | Uganda | Descriptives, generalised estimating equations with robust standard errors |
| **139** | Zhang G et al | Plasma advanced oxidative protein products are associated with anti-oxidative stress pathway genes and malaria in a longitudinal cohort | 2014 | Incidence of malaria infection and disease | 24 months | 312 | Infants born to HIV-negative mothers | Mozambique | Descriptives, principal component analysis, k-means cluster analysis, ANOVA test, independent sample t test, Chi square test, mixed-effects linear regression |
| **140** | Bwayo D et al | Prevalence of glucose-6-phosphate dehydrogenase deficiency and its association with Plasmodium falciparum infection among children in Iganga district in Uganda | 2014 | Prevalence and incidence of Plasmodium falciparum infection, malaria episodes of symptomatic malaria | 12 months | 245 | Children between 6 months and 9 years recruited from community | Uganda | descriptive statistics, Chi square tests, odds ratios, t test, spearman’s correlation coefficient, incidence rates, rate ratios |
| **141** | Kyeyune FX et al | The interaction between malaria and human immunodeficiency virus infection in severely anaemic Malawian children: a prospective longitudinal study | 2014 | Incidence of malaria infections | 18 months | 381 | Severely anaemic children aged between 6 and 60 months | Malawi | Descriptives, Fisher exact test, student’s t test, Mann-Whitney U-test, Pearson’s or spearman’s correlation coefficient, weighted mantel Haenszel test, negative binomial regression |
| **142** | Doumbo S et al | Co-infection of long-term carriers of Plasmodium falciparum with Schistosoma haematobium enhances protection from febrile malaria: a prospective cohort study in Mali | 2014 | Incidence of malaria episodes, time to first or only malaria episode | 7 months | 616 | Children and adults age 3 months to 25 years randomly selected form community | Mali | Descriptives, Fisher exact test, Trend test, Welch t test, likelihood ratio test, Kaplan Meier estimator, Cox regression, log rank test, stratification |
| **143** | Osier FH et al | New antigens for a multicomponent blood-stage malaria vaccine | 2014 | Incidence of malaria episodes | 6 months | 286 | Children up to ten years of age | Kenya | Descriptives, modified Poisson regression, Wilcoxon rank-sum test, Trend test, histograms |
| **144** | Mosha D et al | Effectiveness of intermittent preventive treatment with sulfadoxine-pyrimethamine during pregnancy on placental malaria, maternal anaemia and birthweight in areas with high and low malaria transmission intensity in Tanzania | 2014 | Prevalence of placental parasitaemia | 1 year | 350 | Pregnant women | Tanzania | Descriptives, multiple logistic regression, absolute risk reduction |
| **145** | Gandhi K et al | Variation in the circumsporozoite protein of Plasmodium falciparum: vaccine development implications | 2014 | Time to new infection and new clinical malaria episode | 2 years | 100 | children aged less than 10 years randomly selected from community | Mali | Descriptives, Fisher exact test, Cox regression, multiple logistic regression |
| **146** | Perraut R et al | Association of antibody responses to the conserved Plasmodium falciparum merozoite surface protein 5 with protection against clinical malaria | 2014 | Incidence of malaria attacks | 5.5 months | 611 | Children and adults from community | Senegal | Descriptives, Wilcoxon signed-rank test, spearman rank correlation test, Poisson regression, stratification |
| **147** | Duvignaud A et al | Incidence of malaria-related fever and morbidity due to Plasmodium falciparum among HIV1-infected pregnant women: a prospective cohort study in South Benin | 2014 | Incidence of malaria, parasitemia | 36 weeks | 432 | HIV1-infected pregnant women | Benin | Descriptives, sample size, Fisher exact test, Chi square test, multiple Poisson regression, Wilcoxon rank-sum test |
| **148** | Tran TM et al | Naturally acquired antibodies specific for Plasmodium falciparum reticulocyte-binding protein homologue 5 inhibit parasite growth and predict protection from malaria | 2014 | Incidence of malaria, parasitemia, time to first episode | 7 months | 357 | Healthy children and adults, aged 3 months to 25 years | Mali | Descriptives, Kaplan Meier estimator, log rank test, Andersen-Gill Cox regression with a robust variance estimator, Fisher exact test, Mann-Whitney U test, Wilcoxon rank-sum test, multiple linear regression, log transformation |
| **149** | Gonçalves BP et al | Parasite burden and severity of malaria in Tanzanian children | 2014 | Incidence of malaria, parasitemia, time to first episode of malaria | 4 years | 882 | New-borns | Tanzania | Descriptives, generalised estimating equations, log transformation, student t test, Cox regression |
| **150** | Coulibaly D et al | Stable malaria incidence despite scaling up control strategies in a malaria vaccine-testing site in Mali | 2014 | Incidence of malaria episodes, parasitemia | 4 years | 400 | Children aged 0 to 14 years | Mali | Descriptives, Chi square test, Fisher exact test, multiple Poisson regression |
| **151** | Kangoye DT | Plasmodium falciparum malaria in children aged 0-2 years: the role of foetal haemoglobin and maternal antibodies to two asexual malaria vaccine candidates (MSP3 and GLURP) | 2014 | Incidence of malaria episodes, parasitemia | 24 months | 140 | infants aged between four and six weeks | Burkina Faso | Descriptives, Cox regression, multiple linear regression with Huber-White sandwich estimator, Kaplan Meier estimator, negative binomial regression |
| **152** | Tine RC et al | Feasibility, safety and effectiveness of combining home-based malaria management and seasonal malaria chemoprevention in children less than 10 years in Senegal: a cluster-randomised trial | 2014 | Incidence of malaria episodes | 5 months | 992 | Children <10 years old | Senegal | Descriptives, sensitivity, specificity, positive predictive and negative predictive value, Poisson regression |
| **153** | Tiendrebeogo RW et all | Antibody-dependent cellular inhibition is associated with reduced risk against febrile malaria in a longitudinal cohort study involving Ghanaian children | 2015 | Incidence of clinical malaria, parasitemia | 42 weeks | 797 | Children aged 1–12 years, | Ghana | Descriptives, multiple logistic regression, likelihood ratio test, multiple linear regression, coefficient of variation, Kaplan Meier estimator, log rank, Cox regression, ROC curve, Welch t test, |
| **154** | Degefa T et al | Malaria incidence and assessment of entomological indices among resettled communities in Ethiopia: a longitudinal study | 2015 | Malaria incidence and transmission intensity | 3 months | 604 | Adults >18 years old residing in villages | Ethiopia | Descriptives, ANOVA test, generalized linear mixed-effects regression |
| **155** | Espié E et al | Spatio-Temporal Variations in Malaria Incidence in Children Less than 10 Years Old, Health District of Sokone, Senegal, 2010-2013 | 2015 | Incidence of clinical malaria | 3 years | 1202 | Children under ten years living in rural areas with high Plasmodium falciparum | Senegal | Descriptives, incident rates, time series |
| **156** | Lwanira CN et al | Frequency of RANTES gene polymorphisms and their association with incidence of malaria: a longitudinal study on children in Iganga district, Uganda | 2015 | Incidence of malaria episodes, parasite density | 1 year | 423 | Children aged between 6 months and 9 years recruited from community | Uganda | Descriptives, Chi square test, Wilcoxon rank-sum test, multiple negative binomial regression, Kaplan Meier estimator, log rank test, Cox regression |
| **157** | Stanisic DI et al | Acquisition of antibodies against Plasmodium falciparum merozoites and malaria immunity in young children and the influence of age, force of infection, and magnitude of response | 2015 | Incidence of malaria episodes, parasite density | 16 months | 183 | Children aged 1 to 4 years | Papua New Guinea | Descriptives, Kruskal-Wallis tests or Mann-Whitney U tests, spearman's rank correlation, Chi square tests, a negative binomial regression with generalised estimating equations |
| **158** | Ndungu FM et al | Identifying children with excess malaria episodes after adjusting for variation in exposure: identification from a longitudinal study using statistical count models | 2015 | Number of clinical malaria episodes | 15 years | 2463 | Children up to 15 years | Kenya | Descriptives, multiple Poisson regression, negative binomial, and zero-inflated negative binomial regression, fractional polynomial plots, histograms, time series |
| **159** | Irani V et al | Acquisition of functional antibodies that block the binding of erythrocyte-binding antigen 175 and protection against plasmodium falciparum malaria in children | 2015 | Incidence of asymptomatic reinfection and symptomatic malaria episodes | 6 months | 206 | Malaria-exposed children aged 5–14 years | Papua New Guinean | Descriptives, Cohen kappa coefficient test, Kruskal-Wallis test or Wilcoxon rank-sum tests, Cox regression, Kaplan Meier estimator, log rank test |
| **160** | Laochan N et al | Intervals to Plasmodium falciparum recurrence after anti-malarial treatment in pregnancy: a longitudinal prospective cohort | 2015 | Incidence of recurrent parasitemia, malaria episodes | 63 days | 700 | Pregnant women | Thailand | Descriptives, Chi square test, geometric means or ratios of geometric means, multiple linear regression, robust standard errors using the Huber-White sandwich estimator, Trend test |
| **161** | Daou M et al | Protection of Malian children from clinical malaria is associated with recognition of multiple antigens | 2015 | Incidence of clinical malaria episodes, asymptomatic malaria | 7 months | 99 | Children aged two to 14 years | Mali | Descriptives, Mann-Whitney U test, Kruskal-Wallis with Dunn’s multiple comparison post-hoc test, Wilcoxon signed-rank test, spearman correlation, multiple Poisson, logistic regression, log transformation |
| **162** | Gichohi-Wainaina WN et al | Tumour necrosis factor allele variants and their association with the occurrence and severity of malaria in African children: a longitudinal study | 2015 | Incidence of recurrent malaria episodes | 400 days | 581 | Children aged 6–59 months and with a height-for-age z-score in the range −3 SD to 1.5 SD | Tanzania | Descriptives, Cox regression, histograms, Fisher exact test, Kaplan Meier estimator, peto tests, ANOVA test |
| **163** | Apinjoh TO et al | Determinants of Infant Susceptibility to Malaria During the First Year of Life in South Western Cameroon | 2015 | Incidence of malaria infection episodes | 1 year | 283 | Infants born to women who were positive and negative for malaria | Cameroon | Descriptives, Chi square test, student t test, ANOVA test, Tukey's test, multiple logistic regression |
| **164** | Lopera-Mesa TM et al | Effect of red blood cell variants on childhood malaria in Mali: a prospective cohort study | 2015 | Incidence of malaria episodes, parasite density | 4 years | 1543 | Children aged 6 months to 17 years | Mali | Descriptives, quasi-Poisson regression, log transformation, test for trend, logistic generalised estimating equations |
| **165** | Stanisic DI et al | Risk factors for malaria and adverse birth outcomes in a prospective cohort of pregnant women resident in a high malaria transmission area of Papua New Guinea. | 2015 | Prevalence of malaria infection | 9 months | 328 | Pregnant women | Papua New Guinea | Descriptives, multiple logistic, linear regression, Chi square test, student t test |
| **166** | Yooseph S et al | Stool microbiota composition is associated with the prospective risk of Plasmodium falciparum infection | 2015 | Incidence of symptomatic malaria episodes, parasitemia, time to first malaria episode and time to first infection | 7 months | 695 | Healthy individuals aged 3 months to 25 years | Mali | Descriptives, Kruskal-Wallis, Kaplan Meier estimator, Fisher exact test, log rank test, Cox regression |
| **167** | Harouna AM et al | Effect of cotrimoxazole prophylaxis on the incidence of malaria in HIV-infected children in 2012, in Abidjan, Côte d'Ivoire: a prospective cohort study | 2015 | Incidence of malaria, parasitemia | 6 months | 1117 | HIV-infected children <16 years | Côte d'Ivoire | Descriptives, multiple Poisson regression |
| **168** | Krogstad DJ et al | Molecular incidence and clearance of Plasmodium falciparum infection | 2015 | Incidence and clearance of parasitemia | 12 months | 80 | Children from 6 months to 9 years of age | Mali | Descriptives, Chi square test |
| **169** | Kinung'hi SM et al | The impact of anthelmintic treatment intervention on malaria infection and anaemia in school and preschool children in Magu district, Tanzania: an open label randomised intervention trial | 2015 | Prevalence and incidence of malaria episodes, parasitemia | 24 months | 765 | School children aged 6–13 years, and pre-school children aged 3–5 years | Tanzania | Descriptives, Chi square test, student t test, repeated ANOVA test, relative risk ratio |
| **170** | Alexandre MA et al | The association between nutritional status and malaria in children from a rural community in the Amazonian region: a longitudinal study | 2015 | Incidence of malaria, parasitemia, time to first episode of malaria | 1 year | 248 | Children below 15 years | Brazil | Descriptives, Cox regression, Kaplan Meier estimator, log rank test, Wald test, Chi square test |
| **171** | Travassos MA et al | Hemoglobin c trait provides protection from clinical falciparum malaria in Malian children | 2015 | Incidence of malaria episodes, parasitemia, time to first episode of malaria | 12 months | 300 | Children aged 1–6 years without acute and chronic illnesses | Mali | Descriptives, Cox regression, multiple Poisson regression, generalised estimating equations, Kruskal-Wallis test |
| **172** | Newell K et al | Longitudinal household surveillance for malaria in Rakai, Uganda | 2016 | Incidence of asymptomatic and symptomatic malaria | 12 months | 1459 | Children and adults from randomly selected households | Uganda | Descriptives, time series plot, Chi square test, kappa statistic |
| **173** | Seyoum D et al | Identification of different malaria patterns due to Plasmodium falciparum and Plasmodium vivax in Ethiopian children: a prospective cohort study | 2016 | Incidence of clinical malaria episodes, parasitemia | 96 weeks | 2040 | Children less than 10 years of age | Ethiopia | Descriptives, Cox regression, piecewise Weibull frailty model, Wald test, nested frailty model, martingale residual plots. |
| **174** | Njua-Yafi C et al | Malaria, helminths, co-infection and anaemia in a cohort of children from Mutengene, south western Cameroon | 2016 | Incidence of clinical and malaria parasitaemia | 12 months | 357 | Children aged 6 months to 10 years | Cameroon | Descriptives, log transformation, repeated ANOVA test, Chi square test, multiple logistic regression, Pearson’s correlation coefficient |
| **175** | Kasirye R et al | Longitudinal effect of CD4 by cotrimoxazole use on malaria incidence among HIV-infected Ugandan adults on antiretroviral therapy: a randomized controlled study | 2016 | Incidence of malaria episodes, time to first malaria episode | 2.5 years (median) | 2180 | HIV-infected adults | Uganda | Descriptives, mixed-effects Poisson regression, log transformation |
| **176** | Sylvester B et al | Prenatal exposure to Plasmodium falciparum increases frequency and shortens time from birth to first clinical malaria episodes during the first two years of life: prospective birth cohort study | 2016 | Time to first clinical malaria episode, frequency of clinical malaria episodes | 2 years | 206 | Infants born to mothers without Plasmodium falciparum Parasites in placenta | Tanzania | Descriptives, Chi square test, student t test, log rank test, Kaplan Meier estimator, multiple logistic regression |
| **177** | Kangoye DT et al | Dynamics and role of antibodies to Plasmodium falciparum merozoite antigens in children living in two settings with differing malaria transmission intensity | 2016 | Incidence of malaria episodes, and parasitemia, time to the first episode | 2 years | 150 | Children aged four to six weeks | Burkina Faso, Senegal | Descriptives, Pearson correlation test, log transformation, Fisher exact test, mixed-effects regression, Cox regression, Kaplan Meier estimator, negative binomial regression, Wald test, modified Poisson regression |
| **178** | Kyabayinze DJ et al | HRP2 and pLDH-Based Rapid Diagnostic Tests, Expert Microscopy, and PCR for Detection of Malaria Infection during Pregnancy and at Delivery in Areas of Varied Transmission: A Prospective Cohort Study in Burkina Faso and Uganda | 2016 | Incidence of malaria, recrudescence parasitemia | 11 months | 990 | HIV-uninfected women attending antenatal care | Burkina Faso, Uganda | Descriptives, generalised estimating equations, Fisher exact test, Mann-Whitney U test |
| **179** | De Beaudrap P et al | Timing of malaria in pregnancy and impact on infant growth and morbidity: a cohort study in Uganda | 2016 | Incidence of malaria, time to the first malaria infection | 12 months | 832 | New-borns | Uganda | descriptives, Poisson regression, Trend test |
| **180** | Abdus-Salam RA et al | A comparative study of azithromycin and sulphadoxine-pyrimethamine as prophylaxis against malaria in pregnancy | 2016 | Incidence of parasitemia | 9 months | 200 | Pregnant women attending antenatal clinic | Nigeria | Descriptives, Chi square test, Fisher exact test |
| **181** | Weaver R et al | The association between naturally acquired IgG subclass specific antibodies to the PfRH5 invasion complex and protection from Plasmodium falciparum malaria | 2016 | Incidence of re-infection and symptomatic malaria episodes | 6 months | 206 | Children were aged between 5–14 years | Papua New Guinea | Descriptives, spearman correlation, Kaplan Meier estimator, Cox regression, Chi square test, Kruskal Wallis test, log rank test |
| **182** | Amaratunga C et al | Dihydroartemisinic-piperaquine resistance in Plasmodium falciparum malaria in Cambodia: a multisite prospective cohort study | 2016 | Recrudescent Plasmodium falciparum parasitaemia | 63 days | 241 | Patients aged 2-65 years with uncomplicated P falciparum malaria | Cambodia | Descriptives, Fisher exact test, Kruskal Wallis test, Mann-Whitney U test, Kaplan Meier estimator, Wilcoxon signed-rank test, student t test |
| **183** | Olotu A et al | Seven-Year Efficacy of RTS, S/AS01 Malaria Vaccine among Young African Children | 2016 | Incidence of malaria episodes | 7 years | 447 | Health children who were 5 to 17 months | Kenya, Tanzania | Descriptives, Cox regression, negative binomial regression, Andersen-Gill extension of Cox regression |
| **184** | Kakuru A et al | Malaria burden in a birth cohort of HIV-exposed uninfected Ugandan infants living in a high malaria transmission setting | 2016 | Incidence of malaria episodes, time to first episode of malaria | 12 months | 361 | Infants born to HIV-infected mothers who were part of a randomized controlled trial of lopinavir/ritonavir versus efavirenz-based ART | Uganda | Descriptives, Cox regression, Kaplan Meier estimator |
| **185** | Nash SD et al | A Malaria-Resistant Phenotype with Immunological Correlates in a Tanzanian Birth Cohort Exposed to Intense Malaria Transmission | 2017 | Parasitemia incidence | 2 years | 687 | Infants born to mother with no chronic or debilitating illness | Tanzania | Descriptives, Chi square test, student’s t test, Mann-Whitney, logistic generalised estimating equations, multiple logistic regression, sensitivity analyses |
| **186** | Kana IH et al | Naturally Acquired Antibodies Target the Glutamate-Rich Protein on Intact Merozoites and Predict Protection Against Febrile Malaria | 2017 | Incidence of symptomatic malaria parasite infection | 42 weeks | 798 | Children aged 1–12 years | Ghana | Descriptives, Welch t test, normalization transformation, multiple logistic regression, Kaplan Meier estimator, log rank test, Cox regression, Bonferroni for multiple testing |
| **187** | Dechavanne C et al | Associations between an IgG3 polymorphism in the binding domain for FcRn, transplacental transfer of malaria-specific IgG3, and protection against Plasmodium falciparum malaria during infancy: A birth cohort study in Benin | 2017 | Incidence of symptomatic malaria and asymptomatic malaria episodes | 12 months | 572 | Infants | Benin | Descriptives, Pearson Chi square test, student unpaired t test, multiple logistic regression, Bonferroni correction, mixed-effects linear regression, Kaplan Meier estimator, log rank test, Cox regression, Poisson regression |
| **188** | Tukwasibwe S et al | Drug resistance mediating Plasmodium falciparum polymorphisms and clinical presentations of parasitaemic children in Uganda | 2017 | Incidence of asymptomatic and symptomatic parasitaemia episodes | 3 years | 114 | Aged 4–11 years | Uganda | Descriptives, multiple linear regression, binomial generalised estimating equations |
| **189** | Harrington WE et al | Maternal microchimerism predicts increased infection but decreased disease due to plasmodium falciparum during early childhood | 2017 | Incidence of clinical malaria episodes, parasitemia | 4 years | 53 | Infants born to women with no chronic illness, sickle cell disease, HIV | Tanzania | Descriptives, logistic regression, negative binomial regression, Mann-Whitney U test, binomial and gaussian generalised estimating equations |
| **190** | Adomako-Ankomah Y et al | Host age plasmodium falciparum multiclonality are associated with gametocyte prevalence: a 1-year prospective cohort study | 2017 | Prevalence of malaria infection episodes | 1 year | 500 | Individuals aged 1-65 years | Mali | Descriptives, Mann-Whitney U test, one-way ANOVA test, Tukey's test, Chi square test, histogram, multiple linear regression |
| **191** | Dharmawardena P et al | Response of imported malaria patients to antimalarial medicines in Sri Lanka following malaria elimination. | 2017 | Incidence of malaria episodes, parasitemia | 12 months | 59 | Children and adults | Sri Lanka | Descriptives, Kaplan Meier estimator, Chi square test |
| **192** | Mohapatra PK et al | HIV-malaria interactions in North-East India: A prospective cohort study | 2017 | Incidence of parasitemia | 18 months | 333 | HIV-infected individuals | India | Descriptives, Chi square test |
| **193** | França CT et al | IgG antibodies to synthetic GPI are biomarkers of immune-status to both Plasmodium falciparum and Plasmodium vivax malaria in young children | 2017 | Incidence of malaria episodes, parasitemia | 16 months | 223 | Children aged 1-3 years | Papua New Guinea | Descriptives, generalised estimating equations, student t-test, ANOVA test |
| **194** | Lufele E et al | Risk factors and pregnancy outcomes associated with placental malaria in a prospective cohort of Papua New Guinean women | 2017 | Prevalence of malaria, parasitemia | 9 months | 1451 | Pregnant women | Papua New Guinea | Descriptives, multiple logistic regression, multiple linear regression, Chi square test, student t test |
| **195** | Boudová S Laufer M et al | Placental but not peripheral Plasmodium falciparum infection during pregnancy is associated with increased risk of malaria in infancy | 2017 | Cumulative incidence of infection, clinical malaria | 2 years | 473 | Infants | Malawi | Descriptives, Cox regression Nelson-Aalen cumulative hazard estimate, Kaplan Meier estimator, log rank test, logistic regression, relative rate ratio |
| **196** | Kasirye RP et al | Effect of antiretroviral therapy on malaria incidence in HIV-infected Ugandan adults | 2017 | Incidence of malaria episodes, time to first malaria episode | 2.5 years (median) | 2154 | HIV-infected Ugandan adults | Uganda | Descriptives, Kaplan Meier estimator, log rank test, mixed-effects Poisson regression |
| **197** | Lombardo P et al | Hemoglobin Levels and the Risk of Malaria in Papua New Guinean Infants: A Nested Cohort Study | 2017 | Incidence of malaria episodes, parasitemia | 12 months | 1303 | Children aged 3 (±1) months old at the time of inclusion, no chronic illness, haemoglobin > 5.0 g/dL | Papua New Guinean | Descriptives, Breslow, Cox regression, likelihood ratio test |
